# Modeling Krebs cycle from liver, heart and hepatoma mitochondria, supported Complex I as target for specific inhibition of cancer cell proliferation

**DOI:** 10.3389/fonc.2025.1557638

**Published:** 2025-03-26

**Authors:** Luz Hernández-Esquivel, Isis Del Mazo-Monsalvo, Silvia Cecilia Pacheco-Velázquez, Rocío Daniela Feregrino-Mondragón, Diana Xochiquetzal Robledo-Cadena, Rosina Sánchez-Thomas, Ricardo Jasso-Chávez, Emma Saavedra, Álvaro Marín-Hernández

**Affiliations:** ^1^ Departamento de Bioquímica, Instituto Nacional de Cardiología Ignacio Chávez, Mexico City, Mexico; ^2^ Posgrado en Ciencias Biológicas, Universidad Nacional Autónoma de México, Mexico City, Mexico

**Keywords:** cancer, Krebs cycle, mitochondria, Complex I, heart, liver, kinetic modeling, metabolic control analysis

## Abstract

**Introduction:**

The Krebs cycle (KC) is an important pathway for cancer cells because it produces reduced coenzymes for ATP synthesis and precursors for cellular proliferation. Described changes in cancer KC enzyme activities suggested modifications in the reactions that control the KC flux compared to normal cells.

**Methods:**

In this work, kinetic metabolic models of KC of mitochondria from cancer (HepM), liver (RLM) and heart (RHM) to identify targets to decrease the KC flux were constructed from kinetic parameters (Vmax and Km) of enzymes here determined.

**Results:**

The enzymes Vmax values were higher in the following order: RHM > HepM > RLM; meanwhile, Km values were similar. Kinetic modeling indicated that the NADH consumption reaction (complex I) exerted higher control on the Krebs cycle flux in HepM versus RLM and to a lesser extent in RHM. These results suggested that cancer cells may be more sensitive to complex I inhibition than heart and other non-cancer cells. Indeed, cancer cell proliferation was more sensitive to rotenone (a complex I inhibitor) than heart and non-cancer cells. In contrast, cell proliferation had similar sensitivities to malonate, an inhibitor of succinate dehydrogenase, an enzyme that does not exert control.

**Discussion:**

Our results showed that kinetic modeling and metabolic control analysis allow the identification of high flux-controlling targets in cancer cells that help to design strategies to specifically inhibit their proliferation. This can minimize the toxic effects in normal cells, such as the cardiac ones that are highly sensitive to conventional chemotherapy.

## 1 Introduction

In cancer cells, mitochondria produces approximately 80% of ATP and provides building blocks. Tumor suppressor inactivation or oncogene activation alters mitochondrial metabolism (oxidative phosphorylation, Krebs cycle, fatty acid beta-oxidation, glutamine metabolism, heme metabolism), contributing to cell proliferation, cell survival, metastasis and drug resistance. For this reason, mitochondria are considered a target for designing new therapeutic strategies against cancer (1, 2).

The citric acid or Krebs cycle (KC) is an essential hub in the cell intermediary metabolism; it produces reducing equivalents (NADH and FADH_2_) to synthesize ATP through oxidative phosphorylation (OxPhos); it generates GTP through substrate-level phosphorylation and provides precursors such as oxaloacetate (OAA) for gluconeogenesis, citrate for the synthesis of fatty acids and cholesterol, 2-oxoglutarate (2-oxo) for amino acid anabolism and succinyl CoA for heme biosynthesis (3).

In mitochondria of experimental rat hepatomas, it was initially considered that the Krebs cycle was truncated compared to rat liver mitochondria. In contrast to what is normally expected, oxygen consumption by tumor mitochondria was low when pyruvate (Pyr) was oxidized because citrate produced by citrate synthase (CS) was mainly exported to the cytosol to support the active cholesterol biosynthesis, thus, decreasing citrate availability to oxidation through the conventional KC flux. Such difference in oxygen consumption was not observed when tumor mitochondria were fueled with substrates after citrate synthesis, indicating that KC was complete and functional (4).

Citrate synthase is overexpressed or increases its activity in pancreatic and ovarian cancer (5, 6). In contrast, mitochondrial aconitase (ACO) is downregulated in gastric cancer (7). Furthermore, of the three E1, E2, and E3 subunits that form the 2-oxoglutarate dehydrogenase complex (2OGDH), the E1 subunit (thiamine diphosphate dependent 2-oxo acid dehydrogenase) has splice variants; one of them lacks three regions, one of which is involved in calcium sensitivity and is over-expressed in colorectal cancer according to analysis of genes transcription (8). It has been suggested that the expression of this variant might modify the Ca^2+^ sensitivity of 2OGDH in cancer cells, as occurs with the 2OGDH complex from rat brain mitochondria which contains a greater proportion of E1 Ca^2+^ insensitive subunit (9).

On the other hand, mutations in some enzymes such as succinate dehydrogenase (SDH) and fumarase (FH) have been identified in some types of cancer (renal carcinoma, leukemia, breast cancer); these mutations induce succinate (Suc) or fumarate (Fum) accumulation which in turns promotes prolyl hydroxylases inhibition preventing HIF-1α degradation and hence its stabilization. HIF-1α stabilization promotes increased fluxes of glycolysis and lactate production as end product, thus decreasing carbon flux to pyruvate dehydrogenase (PDH) and KC in mitochondria. As a compensation mechanism, tumor cells increase the use of glutamine to fuel carbon skeletons to the KC at the level of 2OGDH (10). Furthermore, some oncoproteins improve KC functionality in tumor cells; for example, Myc, increases gene expression of the glutamine transporters (ASCT2 and SN2) and glutaminase 1, which together augments glutamine consumption, thus supplying 2-oxo to KC (11, 12).

Increases in the activities of the KC enzymes CS, ACO, isocitrate dehydrogenase (IDH), 2-OGDH, malate dehydrogenase (MDH), besides of PDH have been reported in isolated rat hepatoma mitochondria in comparison to rat liver mitochondria that correlated with an increase in CO_2_ production, a product of KC (13).

The changes in enzyme isoforms expression and enzyme activities in cancer cells suggest possible changes in the steps that exert control on the KC flux compared to non-cancer cells. Metabolic Control Analysis (MCA) permits identifying the main controlling steps in metabolic pathways by determining the flux control coefficients (C^J^_Ei_); this coefficient quantitatively indicates the dependence of a pathway flux on the individual pathway enzyme activities. Among several MCA strategies, kinetic modeling of metabolic pathways permits detailed calculation of the C^J^_Ei_ for each individual pathway enzyme; by obtaining the pathway´s flux control distribution, the steps with higher control on its flux can be proposed as the most promising potential targets to induce flux inhibition (14).

Previous KC kinetic models (15) or oxidative phosphorylation models that included KC have been constructed only for non-tumor cells or tissues such as rat heart mitochondria and neural cells (16–18). Since KC inhibition has been suggested as a therapeutic option in cancer treatment (10) a kinetic model of KC is a platform to determine the control structure of KC in cancer cells and establish the enzymes, transporters or processes that may be inhibited for decreasing the KC flux specifically in cancer cells. This specific targeted approach against cancer cells of a metabolic pathway also present in normal cells is crucial, as standard chemotherapy agents cause several toxic effects on non-cancer cells (19).

In this investigation, KC kinetic models were constructed using kinetic parameters experimentally determined here for that purpose; furthermore, metabolites and fluxes obtained from AS-30D cancer cells mitochondria and non-cancer mitochondria from rat liver and heart were determined to validate the kinetic model predictions. The models helped to identify potential targets and to understand the KC regulatory and controlling mechanisms. From the differences in the kinetic and functional properties of the KC in normal and cancer cells, it may be possible to design strategies to specifically inhibit the energy metabolism in cancer cells without altering or minimizing the effects on the normal cells’ metabolism, in particular heart metabolism that is severed affected by the standard chemotherapy (19).

## 2 Materials and methods

### 2.1 Chemicals

Acetyl-Coenzyme A (Ac-CoA), ADP, coenzyme A (CoA), citrate, CS, DL-isocitrate (DL-Iso), IDH-^NADP^, 2-oxo, Suc, Fum, FH, malate (Mal), MDH, malic enzyme (ME), OAA, thiamine pyrophosphate (TPP), pyridoxal 5- phosphate (PP), NAD^+^, NADH, NADP^+^ and NADPH were purchased from Sigma Chemical (St Louis, MO, USA).

### 2.2 Mitochondria isolation

Rat liver (RLM), rat heart (RHM) and AS-30D rat hepatoma (HepM) mitochondria were prepared as previously described (20–22). All animals were manipulated according to the guidelines NOM-062-ZOO-1999 (Norma Oficial Mexicana) for the care and use of laboratory animals, and the project received approval from the Internal Committee for the Care and Use of Laboratory Animals (CICUAL) with number INC/CICUAL/004/2021 from Instituto Nacional de Cardiología Ignacio Chávez. To decrease contamination by cytosolic proteins, the mitochondrial fractions were resuspended in SHE buffer (250 mM sucrose, 10 mM HEPES, 1 mM EGTA, pH 7.3) and centrifuged at 12857 *x g* for 10 min at 4°C; this process was performed three times. The final pellets were resuspended in SHE buffer plus 1 mM PMSF, 1 mM EDTA and 5 mM DTT at protein concentrations of 30-80 mg/mL and stored at -70°C until use for determination of enzyme activities and Western blotting.

### 2.3 Western blot

Cells were dissolved in RIPA lysis buffer (PBS 1X pH 7.2, 1% IGEPAL NP40, SDS 0.1% and sodium deoxycholate 0.05%) plus 1 tablet of complete protease inhibitors cocktail (Roche; Mannheim, Germany), and mechanically disrupted by passage through an insulin syringe. The lysate was centrifuged at 9600 x g at 4°C for 30 min and the supernatant was recovered and stored at -20°C. Protein samples (40 μg) were resuspended in loading buffer (10% glycerol; 2% SDS and 0.5 M Tris-HCl, 0.002% bromophenol blue, pH 6.8) plus 5% of β-mercaptoethanol and the proteins were separated by SDS-PAGE in 10 or 12.5% polyacrylamide gels under denaturalizing conditions (23). Electrophoretic transfer of proteins to PVDF membranes (BioRad; Hercules, CA, USA) was done as described before (24) followed by overnight immunoblotting at 4°C with antibodies anti-PDH1, anti-CS, anti-IDH2, anti-IDH3, anti-2OGDH, anti-SDH, anti-FH, anti-MDH, anti-ND1 (mitochondrial complex I), anti-COX IV (mitochondrial complex IV), anti-GLUTA (glutaminase), anti-GDH, anti-ME and anti-β-actin at 1:500 dilution. The hybridization bands were revealed with the corresponding secondary antibodies conjugated with horseradish peroxidase (Santa Cruz Biotechnology; Dallas, TX, USA). The signal was detected by chemiluminescence using the ECL-Plus detection system (Amersham Bioscience; Little Chalfont, Buckinghamshire, UK). Densitometry analysis was performed using the Scion Image Software (Scion; Bethesda, MD, USA) and normalized against its respective load control (β-actin).

### 2.4 Determination of enzyme activities and kinetic parameters

The mitochondrial activities of ACO, IDH-^NAD^, IDH-^NADP^, 2OGDH, SDH, FH, MDH, Aspartate aminotransferase (AST), ME, glutamate dehydrogenase (GDH), alanine aminotransferase (ALT) and PDH were determined spectrophotometrically at 340 nm (13, 25) and 37°C, except where indicated. The assay buffer was KME (120 mM KCl, 20 mM MOPS, 1 mM EGTA, pH 7.20) plus 0.02% Triton X-100 and 5 µM rotenone (when the assay was coupled to the production or consumption of NADH).

CS activity was determined as CoA liberation by its thiol reaction with DTNB monitored at 412 nm (13, 25); the standard assay contained 0.1 mM DTNB, 0.003-0.3 mM AcCoA, 0.005-0.2 mM OAA (freshly prepared) and 0.003-0.006 mg of mitochondrial protein. ACO assay contained 1 U IDH-^NADP^, 2 mM MgCl_2_, 0.5 mM NADP^+^, 0.4-1.6 mM citrate and 0.15 mg of mitochondrial protein. IDH-^NAD^ assay contained 0.015-3 mM NAD^+^, 2 mM MgCl_2_, 0.1-8 mM DL-Iso and 0.1 mg of protein. IDH^-NADP^ activity was determined with 0.01-1 mM NADP^+^, 0.1 mM MgCl_2_, 0.015-1.5 mM DL-Iso and 0.1-0.2 mg of mitochondrial protein. The 2-OGDH assay contained 0.15-2 mM NAD^+^, 2 mM MgCl_2_, 1 mM DTT, 1 mM TPP, 0.025-1 mM CoA, 0.75-12 mM 2-oxo and 0.25-0.5 mg of protein. SDH assay contained 0.075 mM DCPIP, 0.4 mM PMS, 0.1-10 mM Suc and 0.025-0.12 mg of protein; the reaction was monitored at 600 nm. The FH reaction assay in the Mal synthesis direction contained 0.5 mM NADP^+^, 1U ME, 2 mM MgCl_2_, 0.1-10 mM Fum (freshly prepared) and 0.03-0.06 mg of protein. The FH reverse reaction assay included 0.08-8 mM Mal and 0.03-0.06 mg of protein, and the formation of the double bond of Fum was detected at 250 nm. MDH assay in the forward reaction included 0.004-0.8 mM NAD^+^, 1U CS, 0.05 mM AcCoA, 0.25-5 mM Mal and 0.04-0.08 mg of protein; the reverse reaction assay contained 0.008-0.1 mM OAA, 0.001-0.1 mM NADH and 0.006-0.012 mg of protein. The ME assay contained 0.1-2.5 mM NADP^+^, 1-15 mM Mal, 1 mM MgCl_2_, and 0.2 mg of protein. GDH assay included 0.1-25 mM glutamate, 0.8 mM MgCl_2_, 2.4 mM ADP, 0.25-10 mM NAD^+^ and 0.05-0.2 mg of protein. GDH reverse reaction assay contained 0.02-0.15 mM NADH, 2.4 mM ADP, 5-120 mM NH_4_Cl, 0.1- 2.5 mM 2-oxo and 0.03-0.125 mg of protein. AST assay contained 0.1-20 mM aspartate, 0.04 mM PP, 0.15 mM NADH, 1U MDH, 0.05-10 mM 2-oxo and 0.02-0.08 mg of protein. ALT assay contained 50 mM alanine, 0.05-10 mM 2-oxo, 0.15 mM NADH, 1U LDH and 0.01-0.025 mg of protein. PDH assay included 0.03-12 mM Pyr, 0.1-10 mM NAD^+^, 0.005-0.4 mM CoA, 1 mM TPP, 1 mM DTT and 0.01 mg of protein.

### 2.5 Determination of metabolite levels and oxygen consumption in isolated mitochondria

To determine levels of KC metabolites, mitochondria (10 mg protein/mL) were incubated at 37°C in KME buffer plus 5 mM Pi, 0.2 mM ADP and one of the following substrates: 2 mM Pyr plus 5 mM Mal, 4 mM glutamine, 10 mM 2-oxo or 5 mM Suc plus 2 µM rotenone for 10 min under orbital shaking at 150 rpm. When incubation was finalized, 5 mM ADP was added; after 3 min, aliquots (1 mL) were withdrawn, mixed with ice-cold KME buffer, and centrifuged at 17,000 *× g* for 1 min at 4°C. The supernatant (extramitochondrial metabolites) was mixed with ice-cold 3% (vol/vol) perchloric acid (PCA) in 1 mM EDTA and kept on ice. The mitochondrial pellet was resuspended in cold KME buffer and centrifuged at 17,000 *× g* for 1 min at 4°C. This procedure was repeated once. The final mitochondrial pellet (intramitochondrial metabolites) was mixed with ice-cold 3% PCA/1 mM EDTA. The supernatant and mitochondrial samples were neutralized with 3 M KOH/0.1 mM Tris and stored at -72°C until used for determination of metabolites by standard enzymatic methods (26).

Mitochondrial respiration (1-2 mg protein/mL) was measured at 37°C using a Clark-type O_2_ electrode in an air-saturated KME medium supplemented with 2 mM Pi. The substrates were 2 mM Pyr plus 5 mM Mal, 4 mM glutamine, 10 mM 2-oxo or 5 mM Suc plus 5 µM rotenone. State 3 respiration was initiated with the addition of 0.3 mM ADP.

### 2.6 Kinetic model construction

The kinetic models of KC of mitochondria of AS-30D, heart and liver included the reactions from CS to MDH besides, the reactions of the anaplerotic enzymes PDH, GDH, ALT, ME and AST. The mitochondrial membrane transport reactions included were pyruvate transport (that represents the pyruvate-proton (Pyr^-^/H^+^) cotransporter) as well as Mal/Suc, Mal/2-oxo and Mal/Iso exchangers (**Supplementary Table S1**). In the models, the CO_2_ concentration was fixed at 2.2 mM, considering CO_2_ dissolved in plasma oscillated between 1.2 and 2.4 mM (27). The CO_2_ concentration was incorporated into the *Keq* value of the equations of the enzymes that produce it (PDH, IDH, 2OGDH) as the ratio Keq*/*[CO_2_] (28). The activity of some enzymes and transporters was parameterized for the simulations to attain the concentrations of metabolites and KC fluxes determined in mitochondria (**Supplementary Table S1**). Concentrations of ADP, Pi, ATP, aspartate, glutamate, NH_4_, GTP, Pyr_out_, Mal_out_, Suc_out_, 2-oxo_out_, coenzyme Q (CoQ), ubiquinol (QH_2_), Iso_out_ and alanine were fixed in the models; these values were here determined or taken from the literature (**Supplementary Table S2**). The model construction and simulations were carried out with COPASI, which also performs MCA and calculates the flux control coefficients from the elasticity coefficients (29). The rate equations used for the KC and anaplerotic enzymes and transporters are as follows:

An ordered reversible ter-bi Michaelis-Menten equation (Equation 1) was used for PDH and 2OGDH kinetics, where A = Pyr or 2-oxo, B= CoA, C= NAD^+^, P= AcCoA or SCoA and Q= NADH. *Ka, Kb, Kc, Kp* and *Kq* are the *Km* values for the corresponding substrates and products, and *Keq* is the equilibrium constant of the reaction.


(1) $$v=\frac{\frac{Vmaxf}{K_{a}K_{b}K_{c}}\left(\left[A\right]\left[B\right]\left[C\right]-\frac{\left[P\right]\left[Q\right]}{K_{eq}}\right)}{1+\frac{\left[A\right]}{K_{a}}+\frac{\left[A\right]\left[B\right]}{K_{a}K_{b}}+\frac{\left[A\right]\left[B\right]\left[C\right]}{K_{a}K_{b}K_{c}}+\frac{\left[P\right]\left[Q\right]}{K_{p}K_{q}}+\frac{\left[Q\right]}{K_{q}}}$$

In RLM and HepM models, PDH and 2OGDH activities were the values reported previously (13) (**Supplementary Table S1**). In RHM model, PDH activity was adjusted 2 times respect to the activity determined (**Supplementary Table S1**) to improve the model prediction of metabolite concentrations and KC flux.

The CS equation (Equation 2) was a random bi-bi reversible Michaelis-Menten equation, where A= AcCoA, B= OAA, P= CoA and Q= Cit. *Ka, Kb, Kp* and *Kq* are the *Km* values for the corresponding substrates and products, and *Keq* is the equilibrium constant of the reaction.


(2) $$v=\frac{\frac{Vmaxf}{K_{a}K_{b}}\left(\left[A\right]\left[B\right]-\frac{\left[P\right]\left[Q\right]}{K_{eq}}\right)}{1+\frac{\left[A\right]}{K_{a}}+\frac{\left[B\right]}{K_{b}}+\frac{\left[A\right]\left[B\right]}{K_{a}K_{b}}+\frac{\left[P\right]}{K_{p}}+\frac{\left[Q\right]}{K_{p}}+\frac{\left[P\right]\left[Q\right]}{K_{p}K_{q}}+\frac{\left[A\right]\left[Q\right]}{K_{a}K_{q}}+\frac{\left[P\right]\left[B\right]}{K_{p}K_{b}}}$$

Mono-reactant reversible equations (Equation 3) described ACO and pyruvate transporter kinetics, where A= Cit or Pyr_out_ and B= Iso or Pyr, *Ka* and *Kb* are the *Km* values for the corresponding substrates and products, respectively, and *Keq* is the equilibrium constant of each reaction.


(3) $$v=\frac{\frac{Vmaxf}{K_{a}}\left(\left[A\right]-\frac{\left[P\right]}{K_{eq}}\right)}{1+\frac{\left[A\right]}{K_{a}}+\frac{\left[P\right]}{K_{p}}}$$

In RLM and HepM models, ACO activity was adjusted in the range determined previously (13) (**Supplementary Table S1**) as well as pyruvate transporter velocity was adjusted in all models to improve the simulation of metabolite concentrations and KC flux (**Supplementary Table S1**).

The rate equation of IDH^-NAD^ was the concerted transition model of Monod-Wyman and Changeux for exclusive ligand binding (isocitrate) together with simple Michaelis-Menten terms for NAD^+^ and reverse reaction (Equation 4). L is the allosteric transition constant. A= NAD^+^, B= Iso, P= 2-oxo and Q= NADH. *Ka, Kb, Kp* and *Kq* are the *Km* values for the corresponding substrates and products, and *Keq* is the equilibrium constant of the reaction (**Supplementary Table S1**).


(4) $$v=Vmaxf\left(\left(\frac{\frac{\left[A\right]}{K_{a}}}{1+\frac{\left[A\right]}{K_{a}}}\right)\left(\frac{{\left[1+\frac{\left[B\right]}{K_{b}}\right]}^{3}}{L{\left(1+\frac{\left[I\right]}{Ki}\right)}^{4}+{\left[1+\frac{\left[B\right]}{K_{b}}\right]}^{4}}\right)-\left(\frac{\frac{\left[P\right]\left[Q\right]}{K_{p}K_{q}K_{eq}}}{1+\frac{\left[P\right]}{K_{p}}+\frac{\left[Q\right]}{K_{q}}+\frac{\left[P\right]\left[Q\right]}{K_{p}K_{q}}}\right)\right)$$

For RLM model, IDH^-NAD^ was adjusted in the range determined (**Table 1**, **Supplementary Table S1**).

**Table 1:** Kinetic parameters of the Krebs cycle and anaplerotic enzymes.

Enzyme		HepM	RLM	RHM
CS	*V_maxf_*	1260 ± 468 (5)*	495 ± 141 (3)	1128 ± 781 (3)
	*Km _OAA_*	0.011 (1)	0.006 (1)	0.007 ± 0.004 (3)
	*Km _AcCoA_*	0.003 (1)	0.005 (1)	0.004 ± 0.003 (3)
ACO	*V_maxf_*	23 ± 8 (3)	13 ± 8 (3)	173 ± 28 (3) #
	*Km _citrate_*	0.26 (2)	0.1 (1)	0.22 ± 0.06 (4)
IDH^-NAD^	*V_maxf_*	125 ± 76 (8)* ^a^	35 ± 14 (4) ^a^	225 ± 129 (4)
	*Km _Iso_*	0.5 (2)	0.15 (2)	1.8 ± 0.4 (4)
	*Km _NAD+_*	0.13 (2)	0.18 (1)	0.2 (1)
	*V_maxf_*	51 ± 17 (6)*	13 ± 4 (6)	N.D.
	*Km I_so_*	5.7 ± 0.02 (3)	3 ± 0.3 (3)	N.D.
	*Ki _NADH vs Iso_*	0.04 ± 0.008 (3)	0.03 ± 0.017 (3)	N.D.
	*n*	2.33 ± 0.33 (3)	1.9 ± 0.5 (3)	N.D.
	*L*	0.64 (2)	1.5 ± 0.75 (3)	N.D.
	*Km _NAD+_*	0.25 ± 0.07 (3)	0.45 ± 0.2 (3)	N.D.
	*Ki _NADH vs NAD+_*	0.08 ± 0.01 (3)	0.05 ± 0.02 (3)	N.D.
2OGDH	*V_maxf_*	22 ± 10 (3)	14 ± 4 (3)	58 ± 21 (3)
	*Km _2-oxo_*	1 ± 0.3 (3)	1.1 ± 0.9 (3)	0.4 ± 0.4 (3)
	*Km _CoA_*	0.015 (1)	0.05 (1)	0.02 ± 0.01 (3)
	*Km _NAD+_*	0.4 ± 0.2 (3)	0.4 ± 0.2 (3)	0.5 ± 0.2 (3)
SDH	*V_maxf_*	13 ± 8 (3)	39 ± 11 (3)	388 ± 352 (3)
	*Km _Suc_*	0.07 (2)	N.D.	1 ± 0.5 (3)
FH	*V_maxf_*	3356 ± 335 (4)*	172 ± 10 (3)	527 ± 209 (3)
	*Km _Fum_*	1 (1)	0.53 (1)	0.26 ± 0.06 (3)
	*V_maxr_*	340 ± 96 (5)	187 ± 78 (3)	523 ± 235 (4)
	*Km _Mal_*	0.4 (1)	1.7 (1)	0.3 ± 0.01 (3)
MDH	*V_maxf_*	269 ± 122 (6)	523 ± 270 (5)	1320 ± 642 (3)
	*Km _Mal_*	0.45 (1)	N.D.	1.4 ± 0.7 (3)
	*Km _NAD+_*	0.11 (1)	N.D.	0.2 ± 0.15 (3)
	*V_maxr_*	2074 ± 831 (7)	1103 ± 518 (3)	666 ± 105 (3)
	*Km _OAA_*	0.007 (1)	0.05 (1)	0.01 (2)
	*Km _NADH_*	0.017 (1)	0.04 (1)	0.02 ± 0.01 (3)
IDH^-NADP^	*V_maxf_*	587 ± 234 (6)*	108 ± 40 (4)	1059 (2)
	*Km _Iso_*	0.046 (1)	0.04 (1)	0.03 (1)
	*Km _NADP+_*	0.078 (1)	N.D.	0.02 (2)
PDH	*V_maxf_*	26 ± 2 (4)*	1.4 ± 0.6 (3)	44 ± 9 (3)
	*Km _Pyr_*	0.21 ± 0.15 (3)	N.D.	0.2 ± 0.040 (3)
	*Km _CoA_*	0.014 ± 0.002 (3)	N.D.	0.04 ± 0.03 (3)
	*Km _NAD+_*	0.34 ± 0.13 (4)	N.D.	0.6 ± 0.4 (3)
AST	*V_maxf_*	527 (2)	417 ± 252 (4)	N.D.
	*Km _2-oxo_*	1.3 (2)	N.D.	N.D.
	*Km _Aspartate_*	0.6 (2)	N.D.	N.D.
ME	*V_maxf_*	6.5 ± 3 (5) ^b^	N.D.	70 ± 26 (3)
	*Km _Mal_*	1.1 ± 0.5 (5)	N.D.	1.4 ± 0.4 (3)
	*Km _NADP+_*	0.4 ± 0.2 (3)	N.D.	0.5 ± 0.3 (3)
GDH	*V_maxf_*	N.D.	N.D.	2 ± 0.8 (3)
	*Km _NADP_*	N.D.	N.D.	0.53 (1)
	*Km _Glu_*	N.D.	N.D.	8.8 (1)
	*V_maxr_*	N.D.	N.D.	19 ± 7 (3)
	*Km _2-oxo_*	N.D.	N.D.	0.08 (1)
ALT	*V_maxf_*	327 ± 269 (3)	56 ± 30 (3)	N. Det.
	*Km _Ala_*	10 ± 6 (3)	9 ± 7 (3)	
	*Km _2-oxo_*	2 ± 1 (3)	1 ± 0.4 (3)	

*V_maxf_* and *V_maxr_* values in nmol/min*mg mitochondrial protein; *Km, Ki, Ka, K_0.5_* values in mM. ^a^ with 2 mM MnCl_2_; ^b^ with 10 mM Fum. *p ≤ 0.05 vs RLM; #p ≤ 0.05 vs HepM using Student’s t-test for non-paired samples. N. D., Not determined; N. Det., Not detected.

The NADP^+^-dependent IDH isoform (IDH^-NADP^) was also incorporated in all models since this enzyme competes with the IDH-^NAD^ for Iso. The initial predictions indicated that IDH-^NADP^ was the main Iso consumer because its *Km _Iso_* value is 100 times lower than that of IDH-^NAD^ (**Table 1**). The IDH-^NADP^ activity is modulated by the ratio NADPH/NADP^+^ and by inhibition of NAD^+^ and GSH (28) (**Supplementary Table S1**). Incorporating GR in the model regulated the GSH/GSSG ratio and partially the NADPH/NADP^+^ ratio, allowing modulation of IDH-^NADP^ activity. IDH^-NADP^ kinetics was described by an ordered bi-bi reversible Michaelis-Menten equation (Equation 5) where A= NADP^+^, B= Iso, P= 2-oxo and Q= NADPH; *Ka, Kb, Kp* and *Kq* are the *Km* values for the corresponding substrates and products, and *Keq* is the equilibrium constant of the reaction. The GSH competitive inhibition *versus* Iso and NAD^+^ competitive inhibition versus NADP^+^ were included (30). *Ki_GSH_* and *Ki_NAD_* are the inhibition constants for GSH and NAD^+^, respectively (**Supplementary Table S1**).


(5) $$v=\frac{\frac{Vmaxf}{K_{a}K_{b}}\left(\left[A\right]\left[B\right]-\frac{\left[P\right]\left[Q\right]}{K_{eq}}\right)}{1+\frac{\left[A\right]}{K_{a}}+\frac{\left[A\right]\left[GSH\right]}{K_{a}Ki_{GSH}}+\frac{\left[P\right]\left[A\right]}{K_{p}K_{a}}+\frac{\left[Q\right]\left[B\right]}{K_{q}K_{b}}+\frac{\left[Q\right]\left[P\right]}{K_{q}K_{p}}+\frac{\left[Q\right]}{K_{Q}}+\frac{\left[NAD^{+}\right]}{Ki_{NAD}}}\;$$

The succinyl-CoA synthetase (SCS) equation was an ordered ter-ter reversible Michaelis-Menten equation, where A= succinyl-CoA (SCoA), B= ADP, C= Pi, P= ATP, Q= CoA and R= Suc. *Ka, Kb, Kc, Kp, Kq* and *Kr* are the *Km* values for the corresponding substrates and products, and *Keq* is the equilibrium constant of the reaction (Equation 6). In HepM model, SCS activity was parameterized because the measured enzyme activity experienced difficulties that were not understood. For RLM and RHM models, SCS activity was adjusted in the range reported (31) and kinetic parameters were taken from the literature (**Supplementary Table S1**). Although there are two isoforms of SCS, ADP and GDP dependent, in the models only the ADP dependent isoform was considered because it participates mainly in the catabolism and GDP dependent in the anabolism (31).


(6) $$v=\frac{\frac{Vmaxf}{K_{a}K_{b}K_{c}}\left(\left[A\right]\left[B\right]\left[C\right]-\frac{\left[P\right]\left[Q\right]\left[R\right]}{K_{eq}}\right)}{1+\frac{\left[A\right]}{K_{a}}+\frac{\left[A\right]\left[B\right]}{K_{a}K_{a}}+\frac{\left[A\right]\left[B\right]\left[C\right]}{K_{a}K_{b}K_{c}}+\frac{\left[P\right]\left[Q\right]\left[R\right]}{K_{p}K_{q}K_{r}}+\frac{\left[Q\right]\left[R\right]}{K_{p}K_{r}}+\frac{\left[R\right]}{K_{r}}}$$

SDH, AST, ALT, ME and substrate transporters (Mal/Iso, Mal/Suc, Mal/2-oxo) kinetics were defined as ordered bi-bi reversible Michaelis-Menten equations (Equation 7). In the case of SDH, A= Suc, B= CoQ, P= QH_2_ and Q= Fum. For ME, A= NADP^+^, B= Mal, P= Pyr and Q= NADPH. The kinetic mechanism of AST and ALT is ping-pong, but its kinetics was simplified by an ordered mechanism, where A= Asp or Ala, B= 2-oxo, P= OAA or Pyr and Q= glutamate. For transporters, A= Mal_out_, B= Iso, 2-oxo or Suc, P= Mal and Q= Iso_out_, 2-oxo_out_ or Suc_out_. *Ka, Kb, Kp* and *Kq* are the *Km* values for the corresponding substrates and products, and *Keq* is the equilibrium constant of the reaction (**Supplementary Table S1**).


(7) $$v=\frac{\frac{Vmaxf}{K_{a}K_{b}}\left(\left[A\right]\left[B\right]-\frac{\left[P\right]\left[Q\right]}{K_{eq}}\right)}{1+\frac{\left[A\right]}{K_{a}}+\frac{\left[A\right]\left[B\right]}{K_{a}K_{b}}+\frac{\left[P\right]\left[Q\right]}{K_{p}K_{q}}+\frac{\left[Q\right]}{K_{q}}}$$

In all models, SDH activity was assumed to be the rate of mitochondrial oxygen consumption in the presence of Suc plus rotenone (**Supplementary Table S1**); SDH activity directly measured was low because an artificial electron acceptor was used in the assay (25). For the RLM model, ME was not included because this enzyme is not present in RLM (32). Meanwhile, the RHM model did not include ALT because its activity was undetected (**Table 1**, **Supplementary Table S1**). The activity of substrate transporters Mal/Iso, Mal/2-oxo and Mal/Suc were parameterized to simulate the concentrations of metabolites and KC fluxes determined in mitochondria and kinetic parameters were taken from the literature (**Supplementary Table S1**). Mal/2-oxo transporter was not included in RLM model because 2-oxo was not detected in the mitochondrial medium incubation when Mal was oxidized.

The FH rate equation was considered a mono-reactant reversible Michaelis-Menten equation (Equation 8), in which A= Fum and P= Mal. *Ka* and *Kp* are the *Km* values for Fum and Mal, respectively (**Supplementary Table S1**).


(8) $$v=\frac{Vmaxf\frac{\left[A\right]}{K_{A}}-Vmaxr\frac{\left[P\right]}{K_{P}}}{1+\frac{\left[A\right]}{K_{A}}+\frac{\left[P\right]}{K_{P}}}\;$$

MDH kinetics was described by an ordered reversible Michaelis-Menten equation (Equation 9) where A= NAD^+^, B= Mal, P= OAA and Q= NADH. *Ka, Kb, Kp* and *Kq* are the *Km* values for the corresponding substrates and products (**Supplementary Table S1**).


(9) $$v=\frac{Vmaxf\frac{\left[A\right]\left[B\right]}{K_{a}K_{b}}-Vmaxr\frac{\left[P\right]\left[Q\right]}{K_{p}K_{q}}}{1+\frac{\left[A\right]}{K_{a}}+\frac{\left[A\right]\left[B\right]}{K_{a}K_{b}}+\frac{\left[P\right]\left[Q\right]}{K_{p}K_{q}}+\frac{\left[Q\right]}{K_{q}}}$$

Glutamate dehydrogenase (GDH) kinetics (Equation 10) was described by simple Michaelis-Menten terms for NADP^+^ and glutamate and reverse reaction for NADPH and 2-oxo together with the concerted transition model of Monod-Wyman and Changeux for exclusive ligand binding for NH_4_^+^ (33). L is the allosteric transition constant; *Ka_ADP_* is the activation constant for ADP, and *Ki_GTP_* is the inhibition constant for GTP. *Ka, Kb, Kp*, *Kq* and *Kr* are the *Km* values for the corresponding substrates and products (**Supplementary Table S1**).


(10) $$\begin{array}{c} v=Vmaxf\left(\frac{\frac{\left[A\right]\left[B\right]}{K_{a}K_{b}}}{1+\frac{\left[A\right]}{K_{a}}+\frac{\left[A\right]\left[B\right]}{K_{a}K_{b}}}\right)\\ -Vmaxr\left(\frac{\frac{\left[P\right]\left[Q\right]}{K_{p}K_{q}}}{1+\frac{\left[P\right]}{K_{p}}+\frac{\left[P\right]\left[Q\right]}{K_{p}K_{q}}}\right)\left(\frac{\frac{\left[R\right]}{K_{r}}{\left[1+\frac{\left[R\right]}{K_{r}}\right]}^{n-1}}{\frac{L{\left(1+\frac{\left[GTP\right]}{Ki_{GTP}}\right)}^{n}}{\left(1+\frac{\left[ADP\right]}{Ka_{ADP}}\right)}+{\left[1+\frac{\left[R\right]}{K_{r}}\right]}^{n}}\right) \end{array}$$

GR was incorporated as an NADPH consumption system, permitting NADPH/NADP^+^ concentrations to be free variable; indeed, GR is the main NADPH consuming reaction in the models. The GR kinetics were described with an ordered Bi-Ter Michaelis-Menten equation (Equation 11). Where A= NADPH, B= GSSG, P= NADP^+^, Q= GSH and R= GSH. *Ka, Kb, Kp*, *Kq* and *Kr* are the *Km* values for the corresponding substrates and products (28) (**Supplementary Table S1**).


(11) $$v=\;\frac{\frac{Vmaxf}{KaKb}\left(\left[A\right]\left[B\right]-\frac{\left[P\right]\left[Q\right]\left[R\right]}{Keq}\right)\;}{1+\frac{\left[A\right]}{Ka}+\frac{\left[A\right]\left[B\right]}{KaKb}+\frac{\left[P\right]\left[Q\right]\left[R\right]}{KpKqKr}+\frac{\left[Q\right]\left[R\right]}{KqKr}+\frac{\left[Q\right]}{Kq}+\frac{\left[R\right]}{Kr}}$$

To maintain the GSSG concentration available to GR, a GSH oxidation reaction was included that represents the glutathione peroxidase reaction and non-enzymatic reaction of GSH with ROS. In addition, a reaction of NADH consumption was incorporated that represents complex I of the respiratory chain. Both reactions were included as irreversible constant fluxes. These fluxes were parameterized to simulate the concentrations of metabolites and KC fluxes determined in mitochondria (**Supplementary Table S1**).

### 2.7 Effect of metabolic inhibitors on cellular proliferation

Human cervix cancer HeLa and SiHa, human prostate cancer PC3, human colon cancer colo-205, and rat heart myoblast H9c2 cells (CRL-1446, ATCC, Manassas, VA, USA) were cultured in DMEM medium, while human breast cancer MCF7 cells were cultivated in MEM medium. Human breast epithelial MCF-10A cells (CRL-10317, ATCC, Manassas, VA, USA) were cultivated in F-12/DMEM medium. All media were supplemented with 25 mM glucose, 10% fetal bovine serum (Biowest, Riverside, MO, USA) and 10,000 U streptomycin/penicillin/mL under an atmosphere of 5% CO_2_ and 95% air at 37°C until 80-90% confluence. All human cancer cell lines were genotyped by the Instituto Nacional de Medicina Genómica (INMEGEN, Mexico City, Mexico) and the analysis showed that the cell lines used shared 87-100% alleles reported by the ATCC (American Type Culture Collection, Manassas, VA, USA) original clones for their authentication.

The cell lines (20 × 10^3^ cells/well) were grown in 96-well plates containing Dulbecco’s Modified Eagle’s Medium (DMEM) (Sigma-Aldrich, St Louis, MO, USA) for 24 h. Afterward, the cells were exposed to rotenone (1 nM to 50 µM) or malonate (0.1µM to 100 mM) for 24 h. Drug effects on cell proliferation were determined by using the 3-(4,5-dimethyl-thiazol-2-yl)-2,5-diphenyltetrazolium bromide (MTT) (Sigma-Aldrich, St Louis, MO, USA) assay. Plates were incubated at 37°C for 3 h; after, the medium was discarded, and the formazan crystals were dissolved with 100 µL DMSO. After 5 min incubation at 37°C, the absorbance was measured at λ 595 nm (34).

### 2.8 Statistical analysis

Analysis was performed using non-paired two tailed Student’s t-test, ANOVA/Tukey or ANOVA and Scheffé *post-hoc* test. p values ≤ 0.05 were considered significant.

## 3 Results

### 3.1 Protein levels of Krebs cycle and anaplerotic enzymes

**Figure 1:**
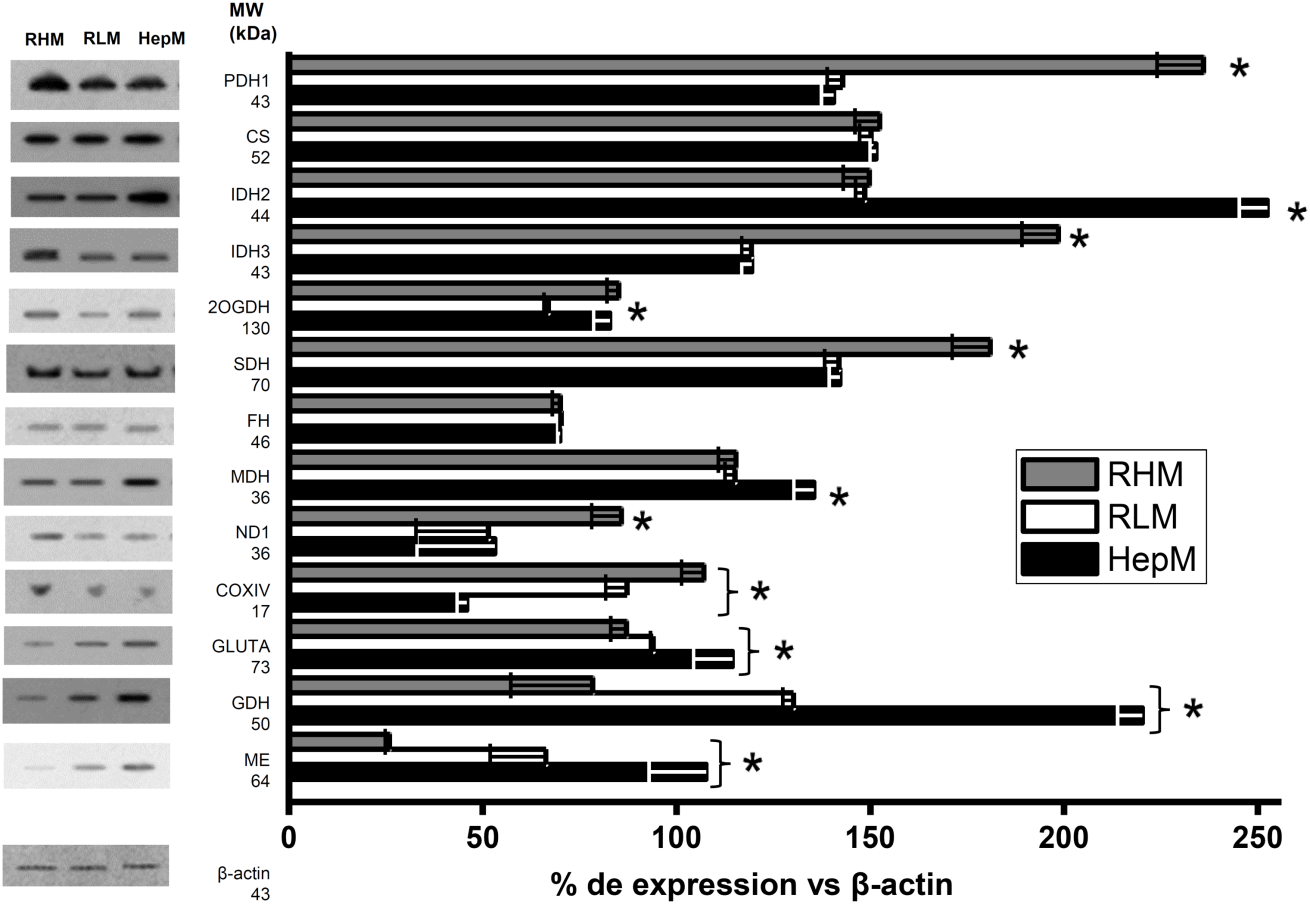
Protein levels of enzymes in isolated mitochondria from heart (RHM), liver (RLM) and AS-30D (HepM) cells. Data are expressed as mean ± standard deviation of 3 independent experiments. All groups were statistically compared using ANOVA and Scheffé *post-hoc* tests with p values < 0.05 as the significance criterion. Original membranes are show in **Supplementary Figure S1**. *p values < 0.05 as the significance criterion.

Western blot analysis showed that the protein levels of pyruvate dehydrogenase (PDH1), isocitrate dehydrogenase 3 (IDH3), succinate dehydrogenase (SDH) and complex I subunits (ND1) are increased 28%- 60% in RHM in comparison to HepM and RLM. Meanwhile, 2OGDH in RLM has lower protein levels (30%) than RHM and HepM. In addition, IDH 2, MDH, GLUTA (glutaminase) and ME content increased between 1.7 to 4.1-folds in HepM compared to RLM and RHM. However, cytochrome c oxidase levels decreased by 48-58% in HepM. In turn, CS and FH had similar levels in the three types of mitochondria (**Figure 1**). These changes in the protein enzyme content suggested differences in enzyme activities among RLM, HepM and RHM.

### 3.2 Krebs cycle and anaplerotic enzyme activities

The kinetic parameters of the Krebs cycle and anaplerotic enzymes were determined in isolated mitochondria. In general, the *Vmax* values of all enzymes showed a tendency to increase (1.7-18-fold) in HepM compared to RLM; with only CS, IDH^-NAD^, FH, IDH^-NADP^ and PDH showing statistically significant differences (**Table 1**). In contrast, the *Vmax* values in RHM were higher (2-31-fold) than in RLM and showed a tendency to increase (1.8-11-fold) compared to HepM; with significant differences observed for ACO, SDH, MDH and ME, IDH^-NADP^. These results suggested that HepM displays more active enzymes than RLM but shows similar or lower levels than heart mitochondria.

The *Km* values of the enzymes in the three types of mitochondria (**Table 1**) were similar and are within the interval reported in Brenda enzyme database (https://www.brenda-enzymes.org).

### 3.3 Krebs cycle metabolites

**Figure 2:**
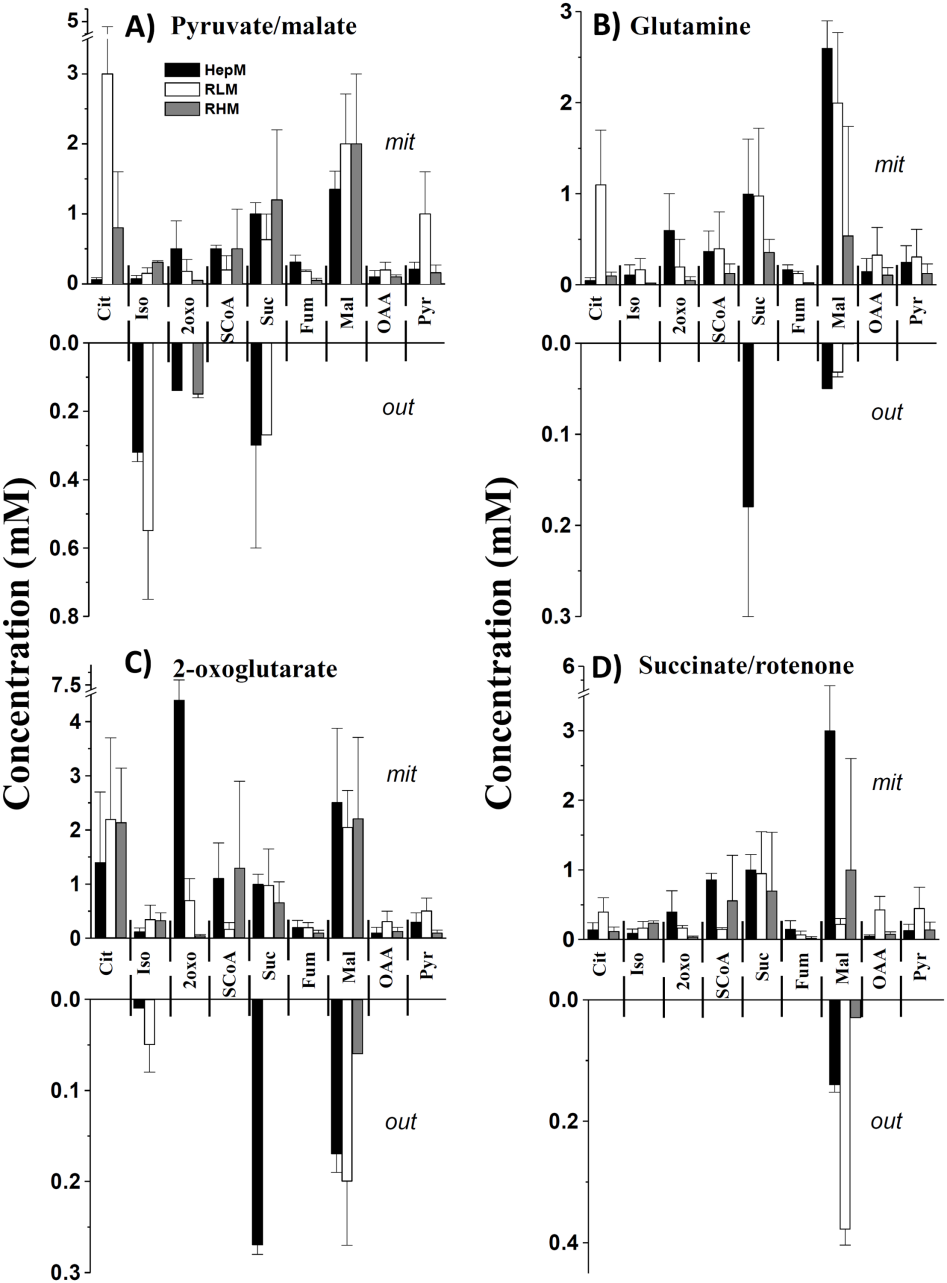
Levels of Krebs cycle metabolites in isolated mitochondria in the presence of different oxidable substrates. The levels of metabolites that were found inside the mitochondria (mit) and released into the incubation medium (out) when **(A)** pyruvate/malate, **(B)** glutamine, **(C)** 2-oxoglutarate or **(D)** succinate/rotenone were used. Data are expressed as mean ± standard deviation of 2-6 independent experiments. Cit, citrate; Iso, isocitrate; 2-oxo, 2-oxoglutarate; SCoA, succinyl-CoA; Suc, succinate; Fum, fumarate; Mal, malate; OAA, oxaloacetate; Pyr, Pyruvate.

The levels of Krebs cycle metabolites were determined in HepM, RLM and RHM incubated with Pyr/Mal, glutamine, 2-oxo or Suc *plus* rotenone (**Figure 2**, **Table 2**). Intramitochondrial KC metabolites concentration varied depending on the oxidizable substrates used, with concentrations increasing in the order of 2-oxo >Pyr/Mal > glutamine > Suc plus rotenone. In RLM, certain metabolites (Cit, Pyr and OAA) showed higher levels than in HepM and RHM, possibly due to the lower activity of Krebs cycle enzymes in RLM (**Figure 2**). The metabolite concentrations observed in the three types of mitochondria were within the range reported for liver, brain and heart mitochondria (35–38).

**Table 2:** Metabolite concentrations and metabolic fluxes determined *in vivo* and simulated by *in silico* modeling for the Pyr-Mal condition.

Metabolite/flux	HepM		RLM		RHM	
	*In vivo*	Model	*In vivo*	Model	*In vivo*	Model
CoA	0.27-2.4*	1.1	0.27-2.4*	0.74	0.27-2.4*	0.73
Pyruvate	0.21± 0.17 (5)	0.23	1 ± 0.6 (4)	1.2	0.16 ± 0.11 (6)	0.15
Acetyl-CoA	0.1-0.86*	0.003	0.1-0.86*	0.003	0.1-0.86*	0.005
Citrate	0.06 ± 0.03 (3)	0.11	3 ± 1.8 (5)	0.2	0.8 ± 0.8 (6)	0.26
Isocitrate	0.07 ± 0.05 (4)	0.13	0.15 ± 0.08 (5)	0.4	0.31 ± 0.02 (5)	0.013
2-oxoglutarate	0.5 ± 0.4 (3)	0.1	0.18 ± 0.17 (4)	0.11	0.05 ± 0.004 (3)	0.015
Succinyl-CoA	0.5 ± 0.05 (3)	0.8	0.2 ± 0.2 (4)	0.85	0.5 ± 0.57 (3)	1.2
Succinate	0.9 ± 0.16 (3)	0.5	0.63 ± 0.37 (4)	0.74	1.2 ± 1 (3)	0.67
Fumarate	0.31 ± 0.1 (3)	0.42	0.18 ± 0.02 (3)	1.2	0.05 ± 0.03 (6)	0.33
Malate	1.35 ± 0.26 (3)	1.41	2 ± 0.71 (3)	2.9	2 ± 1 (6)	0.36
Oxaloacetate	0.1 ± 0.09 (5)	0.07	0.2 ± 0.11 (4)	0.56	0.1 ± 0.03 (5)	0.063
NAD^+^	4-6.4*	5.2	4-6.4*	4.8	4-6.4*	5.1
NADH	0.1-0.3*	0.02	0.1-0.3*	0.4	0.1-0.3*	0.025
NADP^+^	0.5-1.35*	0.33	0.5-1.35*	0.29	0.5-1.35*	0.04
NADPH	0.2-1.2*	1.37	0.5-1.35*	1.4	0.5-1.35*	1.65
GSH	1.6-5*	3.2	2.8-7.4*	5.3	3.4-5*	3.9
GSSG	0.35*	0.39	0.1-0.5*	0.18	0.15-0.55*	0.49
KC-flux	66 ± 1 (4)	65.5	45 ± 3 (4)	45.2	152 ± 4 (4)	153.4

Metabolite concentration in mM; Krebs cycle flux in nmol/min*mg of mitochondrial protein. *Values reported (16, 28, 33, 37, 38).

Metabolite levels in the extramitochondrial incubation medium were determined (**Figure 2**). When Pyr and Mal were used as substrates, Iso, 2-oxo and Suc were observed in the medium. Low levels of Mal were detected with glutamine. Iso, citrate, Suc, and Mal were expelled into the extramitochondrial medium when 2-oxo was oxidized. In the case of Suc oxidation, Mal was detected mainly in the extramitochondrial medium (**Figure 2**). The presence of these metabolites in the extramitochondrial medium might be related to the carriers that transport substrates into the mitochondria.

### 3.4 Rates of oxygen consumption and KC fluxes

**Figure 3:**
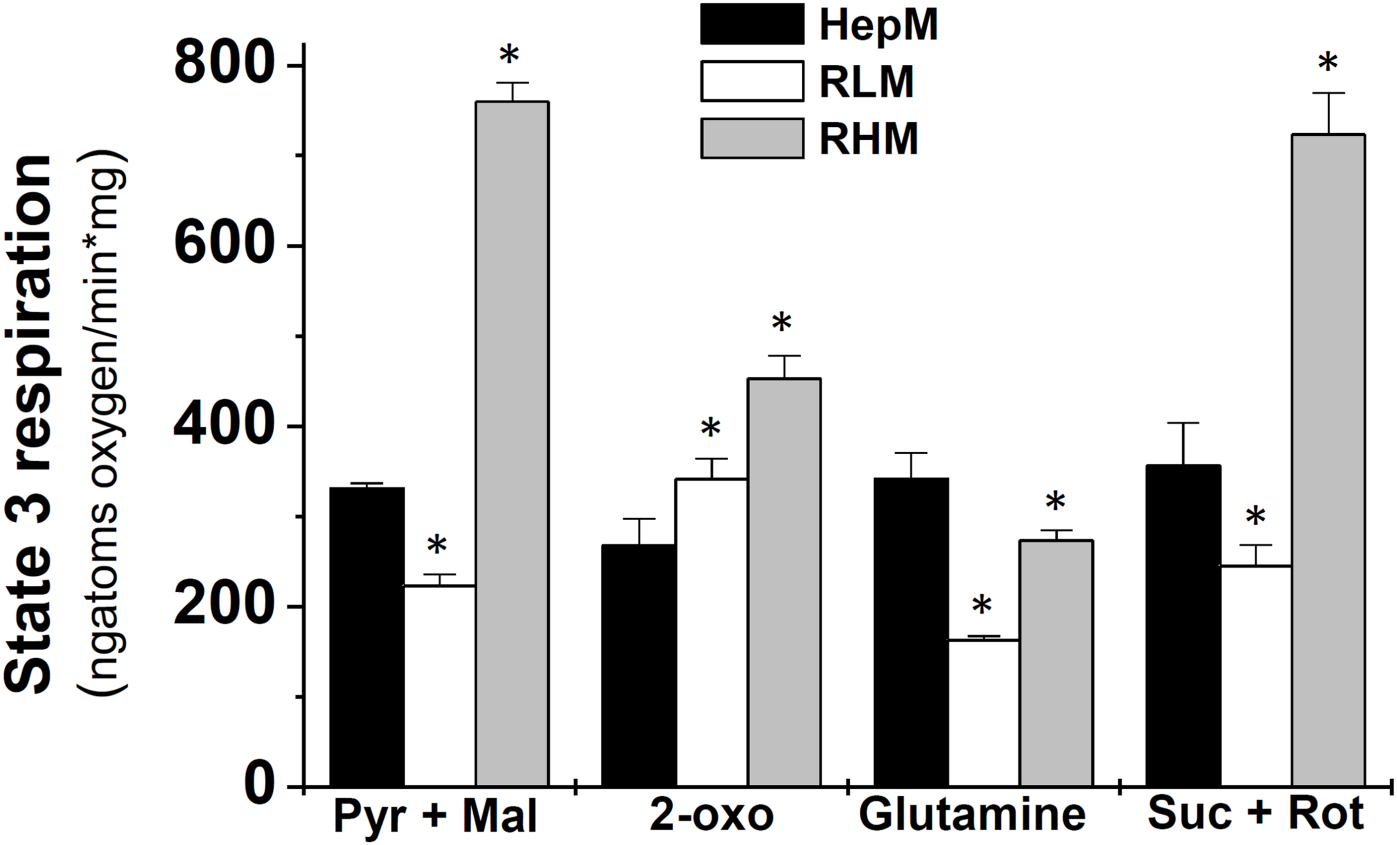
The rates of state 3 respiration in liver, heart and hepatoma mitochondria in the presence of different substrates. Data are expressed as mean ± standard deviation of 4 independent experiments. *p < 0.01 vs. HepM using Student’s t-test for non-paired samples. Pyr, pyruvate; Mal, malate; 2-oxo, 2-oxoglutarate; Suc, succinate; Rot, rotenone.

The mitochondrial oxygen consumption rate is a quantitative measure of the electron transport rate that oxidizes NADH and FADH_2_, products of KC. Therefore, such O_2_-consumption rate was considered an indirect measurement of the KC flux, particularly the O_2_-consumption rate in the presence of ADP (state 3 respiration) that stimulates ATP synthesis. When Pyr/Mal was oxidized, the state 3 respiration in HepM was 49% higher than RLM but 43% lower than in RHM (**Figure 3**). Using 2-oxo as substrate, the state 3 respiration in HepM was 29% and 41% lower than in RLM and RHM, respectively; with glutamine, HepM respiration was 25% and 2-fold higher than RHM and RLM, respectively. Meanwhile, the state 3 respiration of HepM with Suc was 45% higher than RLM but just 49% that of RHM (**Figure 3**).

The Krebs cycle fluxes for each type of mitochondria (**Table 2**) can be estimated considering that each reducing equivalent (NADH and FADH_2_) consumes ½ O_2_ molecule in the electron transport chain (39). Hence, oxidation of Pyr/Mal produces 4 NADH (from PDH, IDH, 2OGDH, MDH) and 1 FADH_2_ (from SDH), therefore a KC turn consumes 2 ½ O_2_ molecules. With this stoichiometry, the KC fluxes estimated were 66 ± 1 (HepM), 45 ± 3 (RLM) and 152 ± 4 (RHM) nmol/min*mg (**Table 2**), indicating that in HepM the KC flux is higher than in its control tissue RLM, but lower than in RHM.

### 3.5 Kinetic models of Krebs cycle

**Figure 4:**
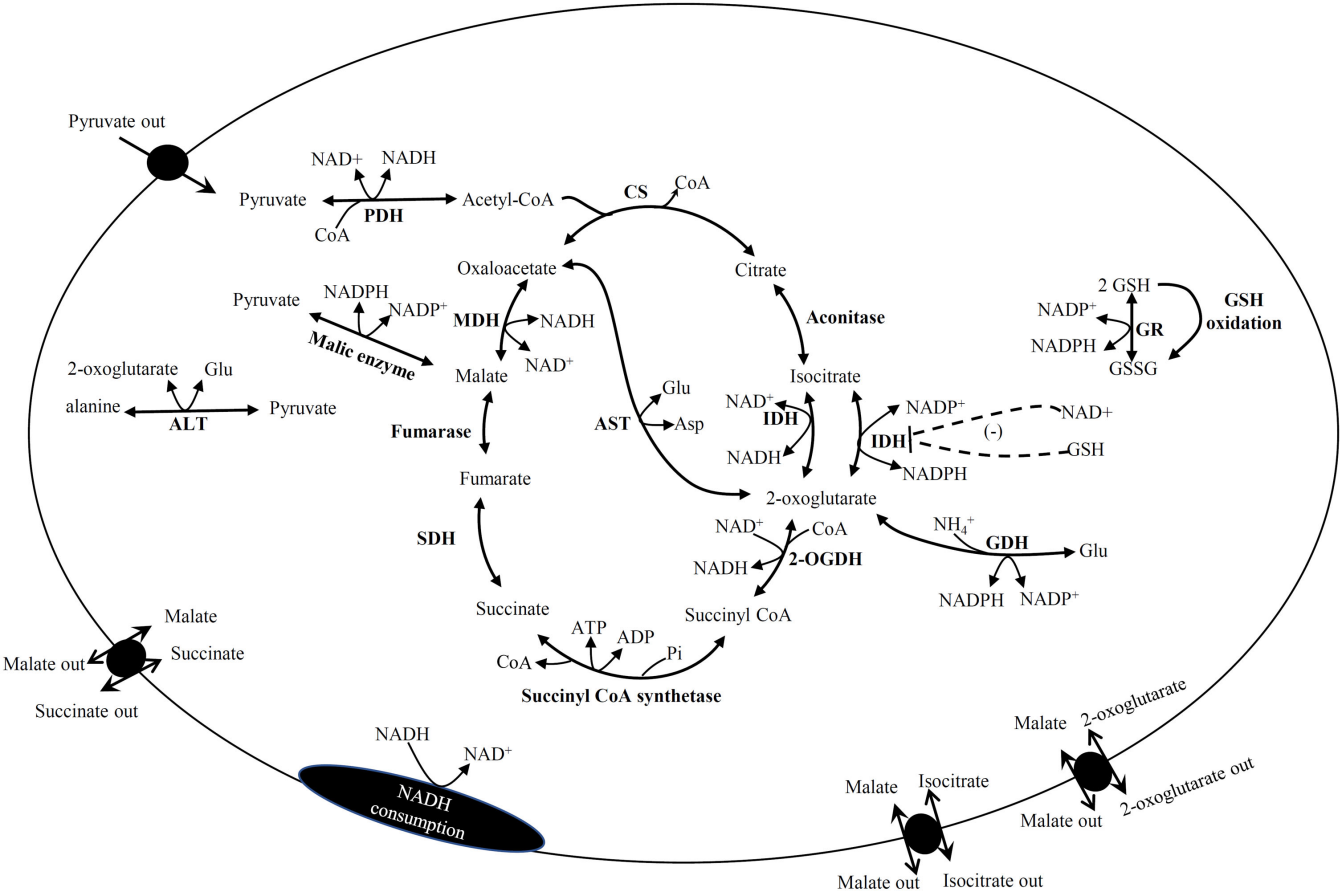
Pathway reactions included in the kinetic models of Krebs cycle. Some reactions were not included in liver and heart. PDH, pyruvate dehydrogenase; CS, citrate synthase; IDH, isocitrate dehydrogenase; 2OGDH, 2-oxoglutarate dehydrogenase; SDH, succinate dehydrogenase; MDH, malate dehydrogenase; ALT, alanine amino transferase; AST, aspartate amino transferase; GR, glutathione reductase; GDH, glutamate dehydrogenase; Glu, glutamate, Asp, aspartate, GSH, reduced glutathione; GSSG, oxidized glutathione.

The kinetic models included the reactions of KC, anaplerotic enzymes (PDH, GDH, ALT, ME and AST), metabolite transporters, and reactions involved in GSH reduction and NADH consumption that represents complex I of the respiratory chain (**Figure 4**). The exceptions were the ME and 2-oxo/Mal transporter which were not included in the RLM model, and the ALT reaction which was not included in the RHM model. In the RHM model, the 2-oxo/Mal exchanger and pyruvate transporter were initially the only ones included in the model. However, the Suc/Mal and Iso/Mal exchangers were later included to modulate the concentrations of Iso and Suc because without these carriers, the first simulations predicted high concentrations of these metabolites.

After an exhaustive refinement process of the kinetic models in a feed-forward working loop that involved experimentation ->model building->simulations ->experimentation -> model refinement, their simulations predicted the majority of metabolites within the same range of concentrations reported or experimentally determined (**Table 2**). The exceptions were the predictions of the levels of AcCoA (in all models), Iso (in RHM), NADH (in HepM and RHM), Fum (in RLM) and NADP^+^ (in RHM), which were two orders of magnitude lower and one order of magnitude higher than the concentrations experimentally determined (**Table 2**).

Regarding the KC fluxes, the one predicted with Pyr/Mal by all models was similar to the flux estimated from the O_2_-consumption rate of state 3 respiration (**Table 2**). In addition, the fluxes through the individual KC enzymes simulated by the models (**Supplementary Table S3**) are in the range previously experimentally determined in RLM and RHM (40, 41). It was considered to analyze only the KC fluxes using Pyr/Mal because in that case all KC enzymes should be working as occurs physiologically with the complete cycle. In contrast, with the other substrates experimentally used such as 2-oxo, Suc/rotenone and glutamine, only partial sections of KC are active during their oxidation.

In general, the kinetic models revealed that AST is a relevant reaction for providing 2-oxo or OAA to keep KC working; these distinguish two main elementary modes comprising CS to IDH and 2OGDH to MDH (**Figure 4**). Interestingly, experimental evidence obtained in rat brain synaptosomes suggested this KC functional organization (42). Furthermore, although IDH2 is not considered a KC component, with its incorporation in the kinetic models, the model predictions suggested that it plays an important role because it is the connection of KC with the antioxidant system by regenerating GSH, an essential metabolite for reactive oxygen species (ROS) detoxification, using the NADPH produced in mitochondria by IDH2 (**Figure 4**).

### 3.6 The flux control distribution of the KC

With the validated kinetic models, the COPASI´s metabolic control analysis tool was used to obtain the concentration and flux control coefficients (**Table 3**). Considering the flux through MDH as the KC flux, the models pointed to the NADH consumption reaction (specifically Complex I) as the main controlling step in the KC of HepM and RHM, being higher in the first. In contrast, the flux control in RLM was shared between 2OGDH and PDH and negatively by the NADH consumption reaction (**Table 3**). This variation in control distribution is attributed to differences in the rates between the reactions of NADH production (PDH, IDH, 2OGDH and MDH) and the NADH consumption reaction. In HepM and RHM, the reaction with lower activity was the NADH consumption reaction; meanwhile, in RLM, it was the NADH production reaction.

**Table 3:** Flux control coefficients (*C^J^_Ei_*) of Krebs cycle and anaplerotic enzymes obtained by kinetic modeling.

	*C^J^_Ei_*		
Pathway step	HepM	RLM	RHM
CS	-5 x 10^-5^	0.001	-6 x 10^-5^
ACO	-5 x 10^-7^	1.4 x 10^-6^	-2 x 10^-6^
IDH^NAD^	-0.24	-0.09	-0.009
2OGDH	**-2.8**	**8.57**	**-0.27**
SCS	-0.02	0.8	-0.016
SDH	-0.01	0.05	-0.004
FH	-0.001	0.01	-3 x 10^-4^
MDH	0.0008	0.02	0.0003
PDH	**-1.9**	**5.43**	**-0.48**
AST	0.0005	0.1	0.0005
ALT	-0.27	-0.15	*
GDH	-0.08	0.24	-9 x 10^-5^
IDH^NADP^	-0.12	-0.14	-0.008
GR	0.0002	2.3 x 10^-5^	6 x 10^-5^
ME	0.31	*	7 x 10^-5^
Pyr Transport	-1.6	0.1	-0.077
Mal-Suc transporter	-0.0009	0.11	-0.009
Mal/2-oxo Transporter	-0.04	*	0.005
Mal-Iso Transporter	0.076	0.008	0.017
GSH oxidation	0.63	-1.6	0.008
NADH consumption	**7.1**	**-12.4**	**1.85**

The *C^J^_Ei_* shown were those obtained for the flux through MDH. The values in boldface type indicate the higher *C^J^_Ei_* of KC enzymes in each mitochondria type. *These reactions do not have values because they were not considered in the corresponding kinetic models.

The flux control coefficients can be positive if an enzyme, transporter, or system favors the pathway flux, while negative does the contrary. In this sense, PDH and 2OGDH have a negative flux control coefficient in HepM and RHM because they compete with MDH for NAD^+^, decreasing the flux through KC (through MDH). In contrast, PDH has a positive flux control coefficient in RLM because its activity promotes the KC flux. Similarly, the flux control coefficient for the NADH consumption reaction in HepM and RHM is positive because it produces NAD^+^, a substrate for PDH, 2OGDH and MDH that favors the KC flux. In contrast, in RLM, the NADH consumption reaction has a negative coefficient because the NAD^+^ production does not stimulate the KC flux.

It is worth noting that in the three kinetic models, the same three high controlling steps were identified 2OGDH, PDH and NADH consumption, either negative or positive, being the latter the reaction with the highest control of all.

### 3.7 The distribution of control of the NADH concentration

In all mitochondria types, the NADH consumption reaction has high negative concentration control coefficient on NADH because it is its substrate (**Table 4**). On the other hand, 2OGDH, PDH and pyruvate transport have high positive concentration control coefficients on NADH in HepM; 2OGDH and PDH in RLM and pyruvate transport in RHM controlling the NADH concentration as part of the NADH producer reactions. Interestingly, in RHM, PDH has a negative concentration control coefficient on NADH because this enzyme maintains a high flux compared to 2OGDH (**Supplementary Table S3**).

**Table 4:** Concentration control coefficients on NADH (*C^NADH^_Ei_*) of the Krebs cycle and anaplerotic enzymes obtained by kinetic modeling.

	*C^NADH^_Ei_*		
Pathway step	HepM	RLM	RHM
CS	0.0008	0.003	-1 x 10^-4^
ACO	7 x 10^-6^	3.9 x 10 ^-6^	3x 10^-6^
IDH^NAD^	0.65	0.08	0.011
2OGDH	**40.3**	**23.6**	**1.12**
SCS	0.28	2.2	0.028
SDH	0.12	0.13	0.006
FH	0.02	0.037	-6 x 10^-4^
MDH	0.54	0.043	**0.51**
PDH	**37.3**	**15**	**-2.2**
AST	0.33	0.21	1.0
ALT	1.83	-0.43	*
GDH	0.59	0.66	0.011
IDH^NADP^	0.83	-0.4	0.98
GR	-0.001	6.4 x 10^-5^	-0.008
ME	-2.1	*	-0.008
Pyr Transport	**10.9**	0.31	**6.6**
Mal-Suc transporter	0.014	0.32	0.016
Mal/2-oxo Transporter	0.68	*	0.06
Mal-Iso Transporter	-0.25	0.004	-0.85
GSH oxidation	-8.5	-4.2	-1.1
NADH consumption	**-83.5**	**-37.6**	**-6.2**

The values in boldface type indicate the higher concentration control coefficients in each mitochondria type. *These reactions do not have values because they were not considered in the corresponding kinetic models.

### 3.8 Effect of complex I inhibition on cellular proliferation

**Figure 5:**
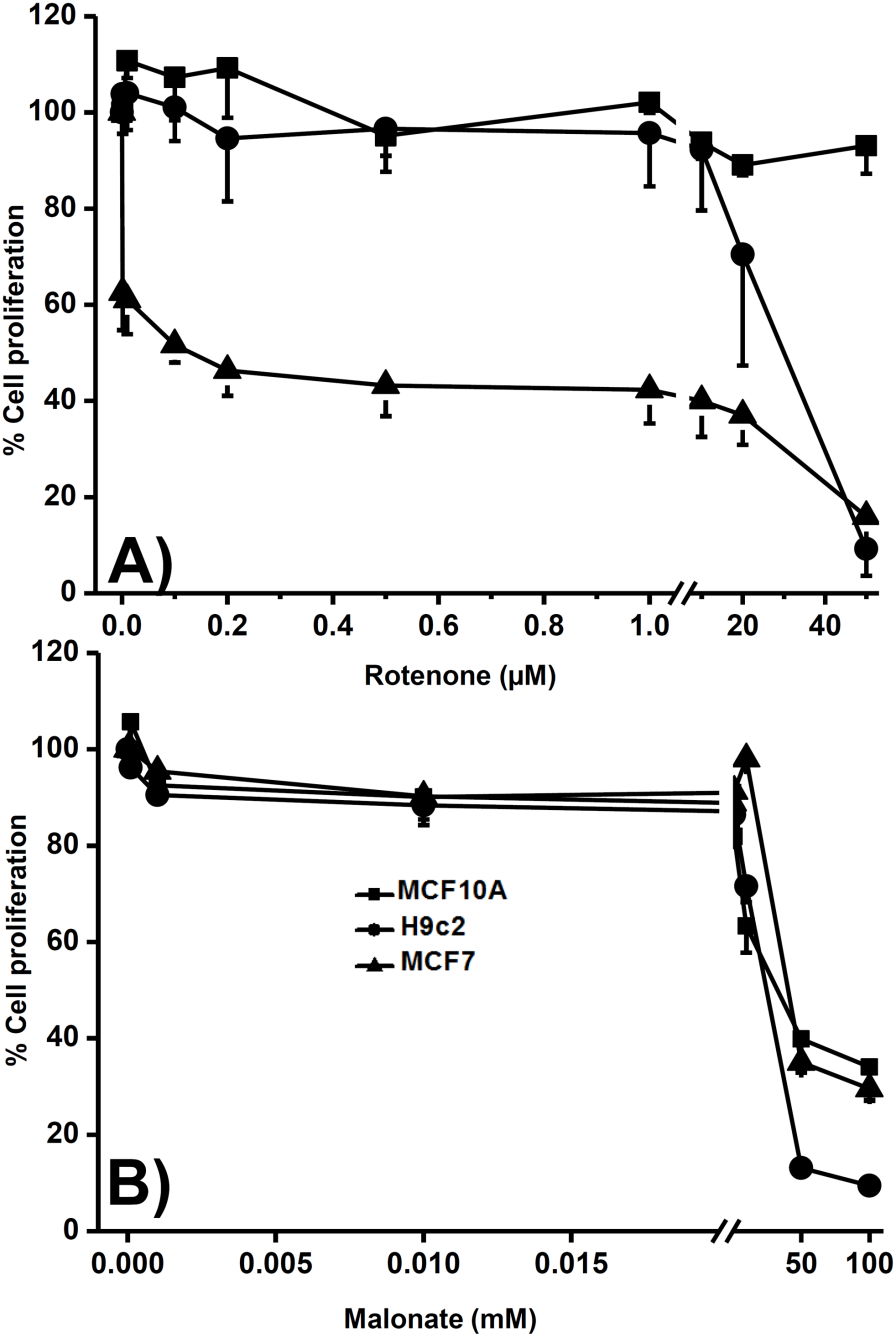
Effect of rotenone and malonate on proliferation of non- and cancer cells. Cells were treated with rotenone from 1 nM to 50 µM **(A)** or malonate from 0.1 µM to 100 mM **(B)** for 24 h. Afterward, proliferation was measured as indicated in the methods. Data are expressed as mean ± standard deviation of 3 independent experiments.

The models predicted that the NADH consumption reaction was the foremost controlling step; as complex I represents the primary site of NADH consumption, rotenone was used as an inhibitor and its effect on cellular proliferation was evaluated in normal and cancer cells. Remarkably, after 24 h of treatment, the proliferation of all cancer cell lines was more sensitive to rotenone than H9c2 cells (heart cells), except for colon cancer cells (COLO-205). In addition, proliferation of breast non-cancer cells (MCF10A) was not affected by the higher concentration of 50 µM rotenone as in MCF7 breast cancer cells (**Table 5**, **Figure 5**). Conversely, non- and cancer cells had similar sensitivity to malonate, an inhibitor of SDH that does not exert control on KC flux (**Table 5**, **Figure 5**). These results suggested that complex I inhibition affected the proliferation mainly in cancer cells, with less effect on heart cells and a null effect on non-cancer cells, which might be attributed to the higher control that complex I exerts on KC in cancer cells.

**Table 5:** IC_50_ values of rotenone and malonate on proliferation of normal and cancer cells.

Cell line	Rotenone (µM)	Malonate (mM)
Non-cancer cell lines		
MCF10A	>50	43 ± 9
H9c2	34 ± 10	25 ± 5
Cancer cell lines		
MCF7	0.15 ± 0.09^a^	41 ± 1
SiHa	18 ± 3^a^	32 (2)
HeLa	17 ± 5^a^	27 ± 5
PC3	13 ± 1^a^	40 ± 2
COLO-205	37 ± 9	32 ± 3

Data shown represent the mean ± S.D. of at least three different preparations. ANOVA/Tukey: ^a^p < 0.05 vs. H9c2.

## 4 Discussion

### 4.1 Distribution of KC enzymes control of mitochondria from different cells

In cancer cells, alterations in gene expression and regulation of KC enzymes modify their activity (10). In this study, the protein levels of KC enzymes did not correlate with the activity, which might be attributed to covalent posttranslational modifications that modulate the enzyme activity, such as phosphorylation, ubiquitination, glycosylation, acetylation, and methylation. For example, acetylation inhibits CS and SDH activity while activate ACO and MDH activity (43).

When comparing cancer mitochondria (HepM) with non-tumoral mitochondria (RLM), an increase in the activity of almost all KC enzymes and anaplerotic enzymes was observed, mainly PDH, IDH-NAD^+^ and 2OGDH, enzymes considered the main controlling steps in normal tissues (13). However, compared to heart mitochondria (RHM), these enzymes showed less activity in HepM, which may be due to the highly oxidative metabolism of heart. The increase in PDH, IDH-NAD^+^, and 2OGDH activity observed in HepM and RHM compared to RLM correlated with an increase in their KC fluxes. These differences already suggested differences in the distribution of control of the KC between these types of mitochondria.

**Figure 6:**
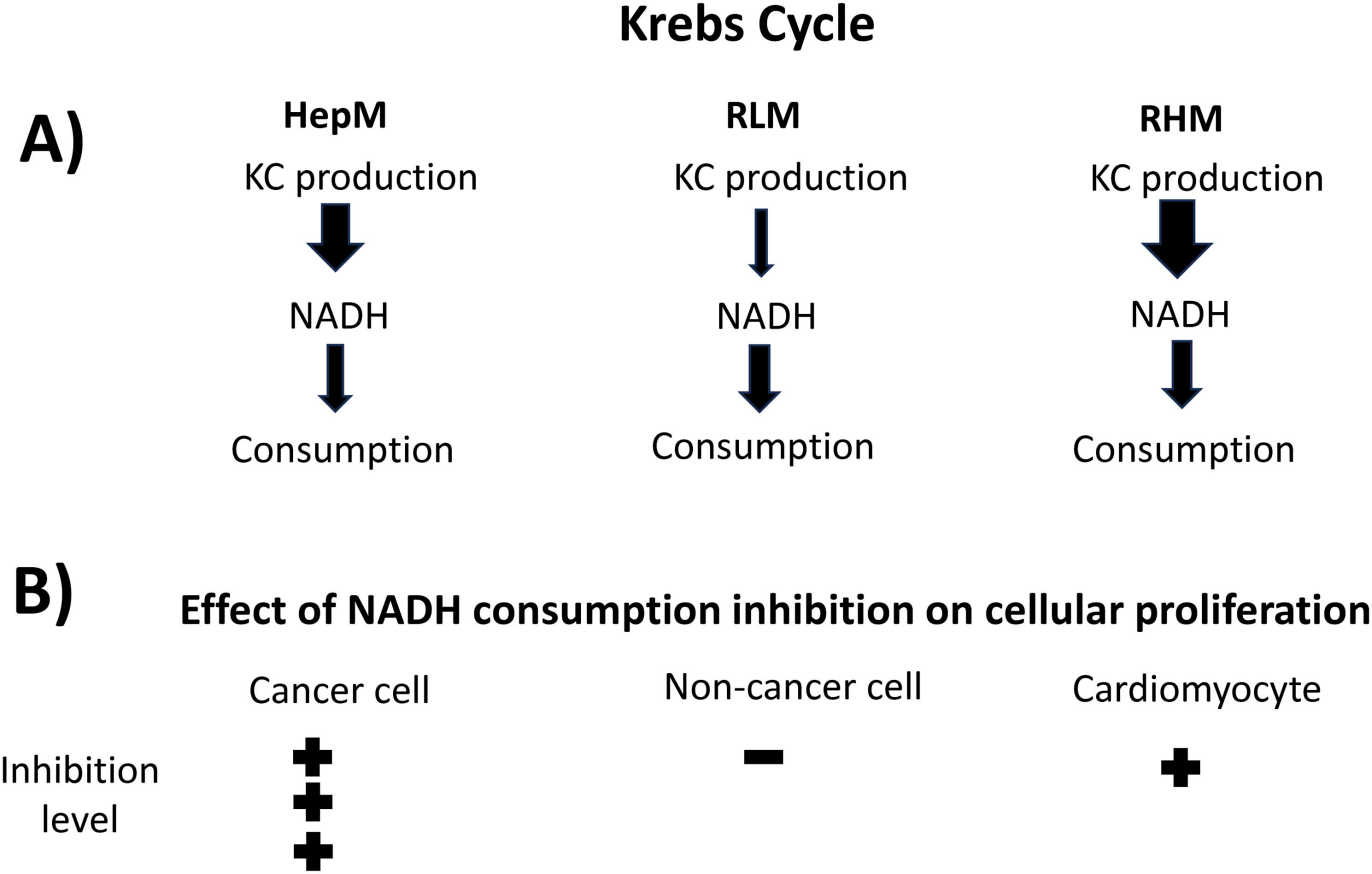
Relation of fluxes of NADH production/consumption and cellular proliferation. **(A)** The variation in flux control distribution is attributed to differences in the rates (arrows) between the reactions of NADH production (PDH, IDH, 2OGDH and MDH) and the NADH consumption reaction (Complex I). Arrow thickness represents rates. **(B)** Inhibition of the NADH consumption reaction with rotenone affected tumor cells in a higher degree than in non-cancer cells due to its higher control of flux.

PDH and 2OGDH, together with CS and IDH-NAD^+^, are generally considered controlling steps of KC flux in non-cancer cells due to their regulatory mechanisms (44, 45). Here, kinetic modeling and MCA indicated that PDH and 2OGDH in RLM and NADH consumption in RHM exert the main control on KC flux. This variation in control distribution between mitochondria of different organs is attributed to differences in the rates between the reactions of NADH production (PDH, IDH, 2OGDH and MDH) and NADH consumption; in RHM, the NADH consumption reaction has lower activity and therefore has higher control, meanwhile, in RLM, the NADH production reactions are the ones with lower activity and therefore the ones with higher control. HepM showed a similar pattern to that of RHM (**Figure 6**). Similar findings in the flux control distribution of KC were reported using the mitochondrial energetics computational model of RHM, where the respiratory chain (NADH consumption) is the main control on KC flux, with the lower contribution of the KC enzymes (46). This difference in KC control structure observed between RLM and RHM has also been reported for oxidative phosphorylation (OxPhos) using the inhibitor method to determine flux control coefficients; OxPhos was mainly controlled by ATP synthase and the phosphate carrier in RLM and by the respiratory chain in RHM, attributed to differences in the activity of the components of OxPhos in each mitochondria type (47). Also, it has been reported that complex I exerts an important control on OxPhos flux in cancer cells compared to non-cancer cells (48), positioning it as a promising target for inhibiting cancer cell proliferation (49).

### 4.2 Specific targeted inhibition in cancer cells based on flux control distribution

In cancer, 2OGDH inhibition has been considered a therapeutic option because cancer cells are 2OGDH-dependent for growth and survival. However, non-cancer cells are also sensitive to 2OGDH inhibition (50–52), which suggests that 2OGDH exerts control on KC flux in non-cancer cells. Indeed, our results demonstrated the high control that 2OGDH has on both cell types, suggesting that 2OGDH inhibition in cancer cells should be tackled with highly specific inhibitors for cancer cells.

On the other hand, Complex I is the principal consumer of the NADH produced by KC and fatty acids β-oxidation, maintaining the NAD^+^ available in the mitochondrial matrix to sustain the activity of the NADH producing enzymes PDH, 2OGDH, IDH and MDH. A halt in NADH oxidation promotes its build up with the consequent KC flux inhibition. The NADH consumption in HepM was the foremost controlling step, even over RHM, suggesting more sensitivity to NADH consumption inhibition in cancer cells. This was demonstrated in the experiments with inhibitors showing that complex I inhibition with rotenone affected more tumor cells than non-cancer cells or highly oxidative cells such as cardiomyocytes (**Figure 6**). This finding is important because several classes of antitumoral agents produce cardiotoxicity that impacts the quality of life of patients (19). Our results suggest a potential strategy to decrease the toxic effects on cardiac and normal cells by inhibiting the high flux-controlling NADH consumption reaction (Complex I) in cancer cells. This is consistent with *in vitro* and *in vivo* findings for several complex I inhibitors (rotenone, metformin, BAY 87-2243, magnolia extract) that have higher negative effects on tumor compared to normal cells (53–57).

The next step is the experimental validation of our findings in *in vivo* tumor models, however, rotenone has the limitation by its neurotoxic effects (58). Notwithstanding, some clinically relevant complex I inhibitors such as metformin, MS-L6 and IACS-010759 have shown low toxicity *in vitro* in mouse liver organoids, rat hepatocytes, non tumoral human cells (lymphocytes, skin fibroblast, lung fibroblast) and murine embryonic fibroblasts in comparison with rotenone (59–61), and their administration inhibits tumor growth in murine xenograft models (human colon cancer, human non-Hodgkin´s B-cell lymphoma cancer, acute myeloid leukemia, glioblastoma and neuroblastoma) without toxicity (56, 59, 61). In addition, some of them and new complex I inhibitors have been used in clinical trials as antitumoral agents, such as metformin (in colorectal, breast and prostate cancer), IACS-010759 (in acute myeloid leukemia and certain solid tumors), IM156 (in gastric, colorectal and ovarian cancer) and MitoTam (in renal cell carcinoma) (62–65).

However, cancer cells can develop resistance to complex I inhibition by metabolic reprogramming. It has been reported that in resistant cells or with deficient complex I activity, glycolysis increase to compensate for mitochondrial dysfunction induced by inactive complex I (66, 67). This metabolic switch might be associated with the activation of HIF1-α, a knowing promoter of glycolysis (67). In addition, the resistant cells maintain an active mitophagy to maintain low levels of complex I (66). Moreover, due to complex I inhibition, ROS production increases and resistant cells can upregulate their antioxidant system, promoting increased ratios of NADPH/NADP^+^ and GSH/GSSG to contend ROS (68). Therefore, multiple inhibitions of controlling steps of other energy metabolism pathways, such as glycolysis and glycogen metabolism, as well as inhibition of the antioxidant system or mitophagy, might be a strategy to avoid the induction of compensatory mechanisms to prevent drug resistance.

### 4.3 Utility of kinetic modeling to understand regulatory mechanisms in metabolic pathways

The results obtained with kinetic modeling and Metabolic Control Analysis validated previous findings on the role of complex I in cell proliferation (53–57). However, as kinetic models are computational simplifications of complex metabolic pathways functions they have some limitations; predictions of some metabolite levels are sometimes outside the range determined or reported, such as occurs here with AcCoA, Iso, NADH, Fum, and NADP+. In the case of AcCoA, mitochondrial endogenous substrates such as fatty acids or amino acids may contribute to the production of AcCoA, reactions that were not considered in the models. Meanwhile, the possible explanation for predictions of the levels of the rest of the metabolites might be the existence of yet unknown regulatory mechanisms of the enzymes involved in producing or consuming these metabolites, which are not considered in the models. Therefore, the kinetic models predictions, help to propose hypotheses that should be experimentally validated (28, 69); in this way, the model serves as a reliable tool for investigating cancer cell metabolism.

Our kinetic model applies to cancer cells that maintain active mitochondria and KC. However, the tumoral environment select populations with different dependences on mitochondria metabolism due to metabolic adaptation and plasticity. For example, some regions of tumors are poorly vascularized, and the supply of oxygen is limited, inducing mitochondria dysfunction. In addition, in some cancer types, mutations in the enzymes of oxidative phosphorylation are reported to limit mitochondrial function. In both cases, cancer cells adapt their energy metabolism, decreasing their dependence on mitochondria with a parallel increase in glycolysis that allows them to survive under these conditions (70). Therefore, the use of combinations of inhibitors of both energy metabolism pathways might be a strategy to decrease cancer populations.

## 5 Conclusion

Kinetic modeling and Metabolic Control Analysis allowed the identification of complex I as a promising target for developing therapeutic strategies to selectively inhibit cancer cell proliferation due to its higher flux control on the Krebs Cycle pathway.

## Data Availability

The original contributions presented in the study are included in the article/**Supplementary Material**. Further inquiries can be directed to the corresponding author.

## References

[1] Ghosh P Vidal C Dey S Zhang L. Mitochondria targeting as an effective strategy for cancer therapy. Int J Mol Sci. (2020) 21:3363. doi: 10.3390/ijms21093363 32397535 PMC7247703

[2] Huang M Myers CR Wang Y You M. Mitochondria as a novel target for cancer chemoprevention: Emergence of mitochondrial-targeting agents. Cancer Prev Res. (2021) 14:285–306. doi: 10.1158/1940-6207.CAPR-20-0425 PMC8137522 33303695

[3] Czibik G Steeples V Yavari A Ashrafian H. Citric acid cycle intermediates in cardioprotection. Circ Cardiovasc Genet. (2014) 7:711–9. doi: 10.1161/CIRCGENETICS.114.000220 25518044

[4] Coleman PS Parlo RA. Warburg’s ghost—Cancer’s self-sustaining phenotype: the aberrant carbon flux in cholesterol-enriched tumor mitochondria via deregulated cholesterogenesis. Front Cell Dev Biol. (2021) 9:626316. doi: 10.3389/fcell.2021.626316 33777935 PMC7994618

[5] Schlichtholz B Turyn J Goyke E Biernacki M Jaskiewicz K Sledzinski Z. Enhanced citrate synthase activity in human pancreatic cancer. Pancreas. (2005) 30:99–104. doi: 10.1097/01.mpa.0000153326.69816.7d 15714131

[6] Chen L Liu T Zhou J Wang Y Wang X Di W. Citrate synthase expression affects tumor phenotype and drug resistance in human ovarian carcinoma. PloS One. (2014) 9:1–19. doi: 10.1371/journal.pone.0115708 PMC4278743 25545012

[7] Wang P Mai C Wei YL Zhao JJ Hu YM Zeng ZL. Decreased expression of the mitochondrial metabolic enzyme aconitase (ACO2) is associated with poor prognosis in gastric cancer. Med Oncol. (2013) 30:552. doi: 10.1007/s12032-013-0552-5 23550275

[8] Snezhkina AV Krasnov GS Zaretsky AR Zhavoronkov A Nyushko KM Moskalev AA. Differential expression of alternatively spliced transcripts related to energy metabolism in colorectal cancer. BMC Genomics. (2016) 17:1011. doi: 10.1186/s12864-016-3351-5 28105922 PMC5249009

[9] Denton RM Pullen TJ Armstrong CT Heesom KJ Rutter GA. Calcium-insensitive splice variants of mammalian E1 subunit of 2-oxoglutarate dehydrogenase complex with tissue-specific patterns of expression. Biochem J. (2016) 473:1165–78. doi: 10.1042/BCJ20160135 PMC6101200 26936970

[10] Anderson NM Mucka P Kern JG Feng H. The emerging role and targetability of the TCA cycle in cancer metabolism. Protein Cell. (2018) 9:216–37. doi: 10.1007/s13238-017-0451-1 PMC5818369 28748451

[11] Wise DR Deberardinis RJ Mancuso A Sayed N Zhang XY Pfeiffer HK. Myc regulates a transcriptional program that stimulates mitochondrial glutaminolysis and leads to glutamine addiction. Proc Natl Acad Sci U.S.A. (2008) 105:18782–7. doi: 10.1073/pnas.0810199105 PMC2596212 19033189

[12] Gao P Tchernyshyov I Chang TC Lee YS Kita K Ochi T. C-Myc suppression of miR-23a/b enhances mitochondrial glutaminase expression and glutamine metabolism. Nature. (2009) 458:762–5. doi: 10.1038/nature07823 PMC2729443 19219026

[13] Dietzen DJ Davis EJ. Oxidation of pyruvate, malate, citrate, and cytosolic reducing equivalents by AS-30D hepatoma mitochondria. Arch Biochem Biophys. (1993) 305:91–102. doi: 10.1006/abbi.1993.1397 8342959

[14] Marín-Hernández Á Saavedra E. Metabolic control analysis as a strategy to identify therapeutic targets, the case of cancer glycolysis. BioSystems. (2023) 231:104986. doi: 10.1016/j.biosystems.2023.104986 37506818

[15] Mogilevskaya E Demin O Goryanin I. Kinetic model of mitochondrial Krebs cycle: Unraveling the mechanism of salicylate hepatotoxic effects. J Biol Phys. (2006) 32:245–71. doi: 10.1007/s10867-006-9015-y PMC2651525 19669466

[16] Wu F Yang F Vinnakota KC Beard DA. Computer modeling of mitochondrial tricarboxylic acid cycle, oxidative phosphorylation, metabolite transport, and electrophysiology. J Biol Chem. (2007) 282:24525–37. doi: 10.1074/jbc.M701024200 17591785

[17] Berndt N Bulik S Holzhütter HG. Kinetic modeling of the mitochondrial energy metabolism of neuronal cells: The impact of reduced α-Ketoglutarate dehydrogenase activities on ATP production and generation of reactive oxygen species. Int J Cell Biol. (2012) 2012:757594. doi: 10.1155/2012/757594 22719765 PMC3376505

[18] Cortassa S Aon MA Marbán E Winslow RL O’Rourke B. An integrated model of cardiac mitochondrial energy metabolism and calcium dynamics. Biophys J. (2003) 84:2734–55. doi: 10.1016/S0006-3495(03)75079-6 PMC1201507 12668482

[19] Abdul-Rahman T Dunham A Huang H Bukhari SMA Mehta A Awuah WA. Chemotherapy induced cardiotoxicity: A state of the art review on general mechanisms, prevention, treatment and recent advances in novel therapeutics. Curr Probl Cardiol. (2023) 48:101591. doi: 10.1016/j.cpcardiol.2023.101591 36621516

[20] Moreno-Sanchez R. Regulation of oxidative phosphorylation in mitochondria by external free Ca2+ concentrations. J Biol Chem. (1985) 260:4028–34. doi: 10.1016/s0021-9258(18)89226-2 2858485

[21] Moreno-Sanchez R Hansford RG. Dependence of cardiac mitochondrial pyruvate dehydrogenase activity on intramitochondrial free Ca2+ concentration. Biochem J. (1988) 256:403–12. doi: 10.1042/bj2560403 PMC1135424 2464995

[22] López-Gómez FJ Eugenia-Torres-Márquez M Moreno-Sánchez R. Control of oxidative phosphorylation in AS-30D hepatoma mitochondria. Int J Biochem. (1993) 25:373–77. doi: 10.1016/0020-711X(93)90627-Q 8096469

[23] Gallardo-Pérez JC de Guevara AAL Marín-Hernández A Moreno-Sánchez R Rodríguez-Enríquez S. HPI/AMF inhibition halts the development of the aggressive phenotype of breast cancer stem cells. Biochim Biophys Acta - Mol Cell Res. (2017) 1864:1679–90. doi: 10.1016/j.bbamcr.2017.06.015 28648642

[24] Pacheco-Velázquez SC Robledo-Cadena DX Hernández-Reséndiz I Gallardo-Pérez JC Moreno-Sánchez R Rodríguez-Enríquez S. Energy metabolism drugs block triple negative breast metastatic cancer cell phenotype. Mol Pharm. (2018) 15:2151–64. doi: 10.1021/acs.molpharmaceut.8b00015 29746779

[25] Reisch AS Elpeleg O. Biochemical assays for mitochondrial activity: assays of TCA cycle enzymes and PDHc. Methods Cell Biol. (2007) 80:199–222. doi: 10.1016/S0091-679X(06)80010-5 17445696

[26] Bergmeyer HUI. Methods of enzymatic analysis. Elsevier Sci. (2012). https://books.google.com.mx/books?id=GDd2zYuLpRwC.

[27] Geers C Gros G. Carbon dioxide transport and carbonic anhydrase in blood and muscle. Physiol Rev. (2000) 80:681–715. doi: 10.1152/physrev.2000.80.2.681 10747205

[28] Moreno-Sánchez R Marín-Hernández Á Gallardo-Pérez JC Vázquez C Rodríguez-Enríquez S Saavedra E. Control of the NADPH supply and GSH recycling for oxidative stress management in hepatoma and liver mitochondria. Biochim Biophys Acta - Bioenerg. (2018) 1859:1138–50. doi: 10.1016/j.bbabio.2018.07.008 30053395

[29] Hoops S Gauges R Lee C Pahle J Simus N Singhal M. COPASI - A COmplex PAthway SImulator. Bioinformatics. (2006) 22:3067–74. doi: 10.1093/bioinformatics/btl485 17032683

[30] Moreno-Sánchez R Gallardo-Pérez JC Rodríguez-Enríquez S Saavedra E Marín-Hernández Á. Control of the NADPH supply for oxidative stress handling in cancer cells. Free Radic Biol Med. (2017) 112:149–61. doi: 10.1016/j.freeradbiomed.2017.07.018 28739529

[31] Lambeth DO Tews KN Adkins S Frohlich D Milavetz BI. Expression of two succinyl-CoA synthetases with different nucleotide specificities in mammalian tissues. J Biol Chem. (2004) 279:36621–4. doi: 10.1074/jbc.M406884200 15234968

[32] Moreadith RW Lehninger AL. Purification, kinetic behavior, and regulation of NAD(P)+ Malic enzyme of tumor mitochondria. J Biol Chem. (1984) 259:6222–7. doi: 10.1016/s0021-9258(20)82129-2 6725250

[33] Moreno-Sánchez R Marín-Hernández Á Gallardo-Pérez JC Pacheco-Velázquez SC Robledo-Cadena DX Padilla-Flores JA. Physiological role of glutamate dehydrogenase in cancer cells. Front Oncol. (2020) 10:429. doi: 10.3389/fonc.2020.00429 32328457 PMC7160333

[34] Robledo-Cadena DX Gallardo-Pérez JC Dávila-Borja V Pacheco-Velázquez SC Belmont-Díaz JA Ralph SJ. Non-steroidal anti-inflammatory drugs increase cisplatin, paclitaxel, and doxorubicin efficacy against human cervix cancer cells. Pharmaceuticals. (2020) 13:463. doi: 10.3390/ph13120463 33333716 PMC7765098

[35] Fedotcheva NI Sokolov AP Kondrashova MN. Nonezymatic formation of succinate in mitochondria under oxidative stress. Free Radic Biol Med. (2006) 41:56–64. doi: 10.1016/j.freeradbiomed.2006.02.012 16781453

[36] Kosenko E Felipo V Montoliu C Grisolía S Kaminsky Y. Effects of acute hyperammonemia *in vivo* on oxidative metabolism in nonsynaptic rat brain mitochondria. Metab Brain Dis. (1997) 12:69–82. doi: 10.1007/BF02676355 9101539

[37] Hansford RG Johnson RN. The steady state concentrations of coenzyme A SH and coenzyme A thioester, citrate, and isocitrate during tricarboxylate cycle oxidations in rabbit heart mitochondria. J Biol Chem. (1975) 250:8361–75. doi: 10.1016/s0021-9258(19)40767-9 1194259

[38] Siess EA Brocks DG Wieland OH. Subcellular distribution of key metabolites in isolated liver cells from fasted rats. FEBS Lett. (1976) 69:265–71. doi: 10.1016/0014-5793(76)80701-6 992036

[39] Bartman CR Weilandt DR Shen Y Lee WD Han Y TeSlaa T. Slow TCA flux and ATP production in primary solid tumours but not metastases. Nature. (2023) 614:349–57. doi: 10.1038/s41586-022-05661-6 PMC10288502 36725930

[40] Stucki JW Walter P. Pyruvate metabolism in mitochondria from rat liver: measured and computer-simulated fluxes. Eur J Biochem. (1972) 30:60–72. doi: 10.1111/j.1432-1033.1972.tb02072.x 4263926

[41] LaNoue K Nicklas WJ Williamson JR. Control of citric acid cycle activity in rat heart mitochondria. J Biol Chem. (1970) 245:102–11. doi: 10.1016/s0021-9258(18)63427-1 4312474

[42] Yudkoff M Nelson D Daikhin Y Erecińska M. Tricarboxylic acid cycle in rat brain synaptosomes: Fluxes and interactions with aspartate aminotransferase and malate/aspartate shuttle. J Biol Chem. (1994) 269:27414–20. doi: 10.1016/s0021-9258(18)47001-9 7961653

[43] Marín-Hernández Á Rodríguez-Zavala JS Jasso-Chávez R Saavedra E Moreno-Sánchez R. Protein acetylation effects on enzyme activity and metabolic pathway fluxes. J Cell Biochem. (2022) 123:701–18. doi: 10.1002/JCB.30197 34931340

[44] Sheu KFR Blass JP. The α-ketoglutarate dehydrogenase complex. Ann New York Acad Sci. (1999) 893:61–78. doi: 10.1111/j.1749-6632.1999.tb07818.x 10672230

[45] Arnold PK Finley LWS. Regulation and function of the mammalian tricarboxylic acid cycle. J Biol Chem. (2023) 299:102838. doi: 10.1016/j.jbc.2022.102838 36581208 PMC9871338

[46] Cortassa S O’Rourke B Winslow RL Aon MA. Control and regulation of integrated mitochondrial function in metabolic and transport networks. Int J Mol Sci. (2009) 10:1500–13. doi: 10.3390/ijms10041500 PMC2680629 19468321

[47] Rossignol R Letellier T Malgat M Rocher C Mazat JP. Tissue variation in the control of oxidative phosphorylation: Implication for mitochondrial diseases. Biochem J. (2000) 347:45–53. doi: 10.1042/0264-6021:3470045 10727400 PMC1220929

[48] Puurand M Tepp K Kaambre T. Diving into cancer OXPHOS – The application of metabolic control analysis to cell and tissue research. BioSystems. (2023) 233:105032. doi: 10.1016/j.biosystems.2023.105032 37739307

[49] Urra FA Muñoz F Lovy A Cárdenas C. The mitochondrial Complex(I)ty of cancer. Front Oncol. (2017) 7:118. doi: 10.3389/fonc.2017.00118 28642839 PMC5462917

[50] Allen EL Ulanet DB Pirman D Mahoney CE Coco J Si Y. Differential aspartate usage identifies a subset of cancer cells particularly dependent on OGDH. Cell Rep. (2016) 17:876–90. doi: 10.1016/j.celrep.2016.09.052 27732861

[51] Chang L-C Chiang S-K Chen S-E Hung M-C. Targeting 2-oxoglutarate dehydrogenase for cancer treatment. Am J Cancer Res. (2022) 12:1436–55. PMC9077069 35530286

[52] Bunik V Mkrtchyan G Grabarska A Oppermann H Daloso D Araujo WL. Inhibition of mitochondrial 2-oxoglutarate dehydrogenase impairs viability of cancer cells in a cell-specific metabolism-dependent manner. Oncotarget. (2016) 7:26400–21. doi: 10.18632/oncotarget.8387 PMC5041988 27027236

[53] Palorini R Simonetto T Cirulli C Chiaradonna F. Mitochondrial complex i inhibitors and forced oxidative phosphorylation synergize in inducing cancer cell death. Int J Cell Biol. (2013) 2013:243876. doi: 10.1155/2013/243876 23690779 PMC3638674

[54] Basit F Van Oppen LMPE Schöckel L Bossenbroek HM Van-Emst-De-Vries SE Hermeling JCW. Mitochondrial complex i inhibition triggers a mitophagy-dependent ROS increase leading to necroptosis and ferroptosis in melanoma cells. Cell Death Dis. (2017) 8:e2716. doi: 10.1038/cddis.2017.133 28358377 PMC5386536

[55] Zhang Q Cheng G Pan J Zielonka J Xiong D Myers CR. Magnolia extract is effective for the chemoprevention of oral cancer through its ability to inhibit mitochondrial respiration at complex i. Cell Commun Signal. (2020) 18:58. doi: 10.1186/s12964-020-0524-2 32264893 PMC7140380

[56] Wheaton WW Weinberg SE Hamanaka RB Soberanes S Sullivan LB Anso E. Metformin inhibits mitochondrial complex I of cancer cells to reduce tumorigenesis. Elife. (2014) 2014:e02242. doi: 10.7554/eLife.02242 PMC4017650 24843020

[57] Martin SD McGee SL. A systematic flux analysis approach to identify metabolic vulnerabilities in human breast cancer cell lines. Cancer Metab. (2019) 7:12. doi: 10.1186/s40170-019-0207-x 31890204 PMC6935091

[58] Guo Z Ruan Z Zhang D Liu X Hou L Wang Q. Rotenone impairs learning and memory in mice through microglia-mediated blood brain barrier disruption and neuronal apoptosis. Chemosphere. (2022) 291:132982. doi: 10.1016/j.chemosphere.2021.132982 34822863

[59] Molina JR Sun Y Protopopova M Gera S Bandi M Bristow C. An inhibitor of oxidative phosphorylation exploits cancer vulnerability. Nat Med. (2018) 24:1036–46. doi: 10.1038/s41591-018-0052-4 29892070

[60] Yang Q Wang L Liu J Cao W Pan Q Li M. Targeting the complex I and III of mitochondrial electron transport chain as a potentially viable option in liver cancer management. Cell Death Discovery. (2021) 7:293. doi: 10.1038/s41420-021-00675-x 34650055 PMC8516882

[61] Al Assi A Posty S Lamarche F Chebel A Guitton J Cottet-Rousselle C. A novel inhibitor of the mitochondrial respiratory complex I with uncoupling properties exerts potent antitumor activity. Cell Death Dis. (2024) 15:1–14. doi: 10.1038/s41419-024-06668-9 38697987 PMC11065874

[62] Yap TA Daver N Mahendra M Zhang J Kamiya-Matsuoka C Meric-Bernstam F. Complex I inhibitor of oxidative phosphorylation in advanced solid tumors and acute myeloid leukemia: phase I trials. Nat Med. (2023) 29:115–26. doi: 10.1038/s41591-022-02103-8 PMC11975418 36658425

[63] Janku F Beom SH Moon YW Kim TW Shin YG Yim DS. First-in-human study of IM156, a novel potent biguanide oxidative phosphorylation (OXPHOS) inhibitor, in patients with advanced solid tumors. Invest New Drugs. (2022) 40:1001–10. doi: 10.1007/s10637-022-01277-9 PMC9395488 35802288

[64] Bielcikova Z Stursa J Krizova L Dong L Spacek J Hlousek S. Mitochondrially targeted tamoxifen in patients with metastatic solid tumours: an open-label, phase I/Ib single-centre trial. eClinicalMedicine. (2023) 57:101873. doi: 10.1016/j.eclinm.2023.101873 37064512 PMC10102891

[65] Greene J Segaran A Lord S. Targeting OXPHOS and the electron transport chain in cancer; Molecular and therapeutic implications. Semin Cancer Biol. (2022) 86:851–9. doi: 10.1016/j.semcancer.2022.02.002 35122973

[66] Ezrova Z Nahacka Z Stursa J Werner L Vlcak E Kralova Viziova P. SMAD4 loss limits the vulnerability of pancreatic cancer cells to complex I inhibition via promotion of mitophagy. Oncogene. (2021) 40:2539–52. doi: 10.1038/s41388-021-01726-4 33686239

[67] Shi Y Wang Y Jiang H Sun X Xu H Wei X. Mitochondrial dysfunction induces radioresistance in colorectal cancer by activating [Ca2+]m-PDP1-PDH-histone acetylation retrograde signaling. Cell Death Dis. (2021) 12:837. doi: 10.1038/s41419-021-03984-2 34489398 PMC8421510

[68] Zhang L Zhang J Ye Zw Muhammad A Li L Culpepper JW. Adaptive changes in tumor cells in response to reductive stress. Biochem Pharmacol. (2024) 219:115929. doi: 10.1016/j.bcp.2023.115929 38000559 PMC10895707

[69] Marín-Hernández Á Gallardo-Pérez JC Reyes-García MA Sosa-Garrocho M Macías-Silva M Rodríguez-Enríquez S. Kinetic modeling of glucose central metabolism in hepatocytes and hepatoma cells. Biochim Biophys Acta - Gen Subj. (2020) 1864:129687. doi: 10.1016/j.bbagen.2020.129687 32712171

[70] Fendt SM Frezza C Erez A. Targeting metabolic plasticity and flexibility dynamics for cancer therapy. Cancer Discovery. (2020) 10:1797–807. doi: 10.1158/2159-8290.CD-20-0844 PMC7710573 33139243

