# Supplementary material for: Modeling Krebs cycle from liver, heart and hepatoma mitochondria, supported Complex I as target for specific inhibition of cancer cell proliferation
Source: Front Oncol. 2025 Mar 26;15:1557638. doi: 10.3389/fonc.2025.1557638 (PMC11979947; doi:10.3389/fonc.2025.1557638)

Supplementary Figure S1. Original Western blot membranes

PDH-E1 α 43 kDa

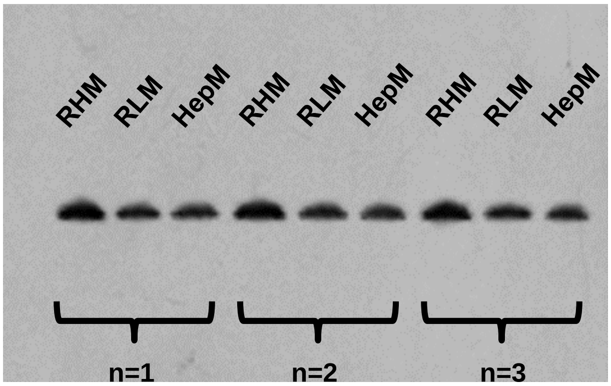

CS 52 kDa

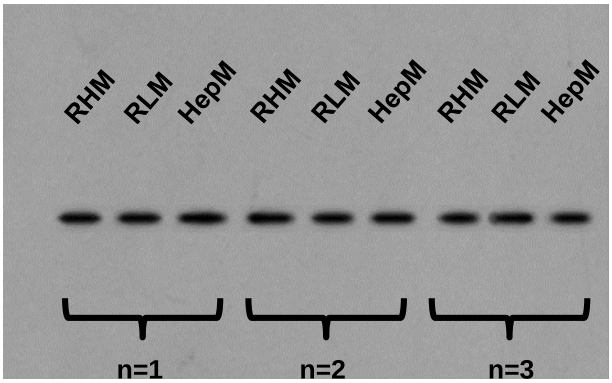

IDH2 44 KDa

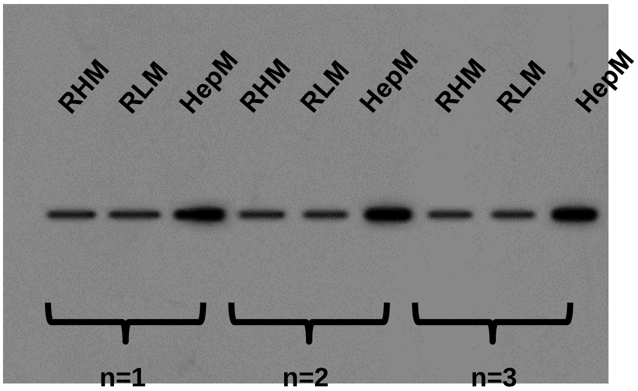

IDH3G 43 KDa

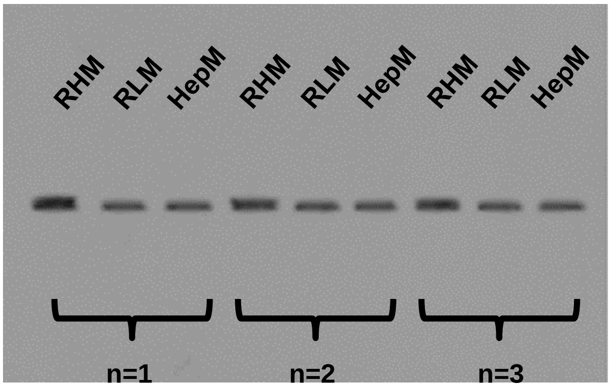

2OGDH 130 kDa

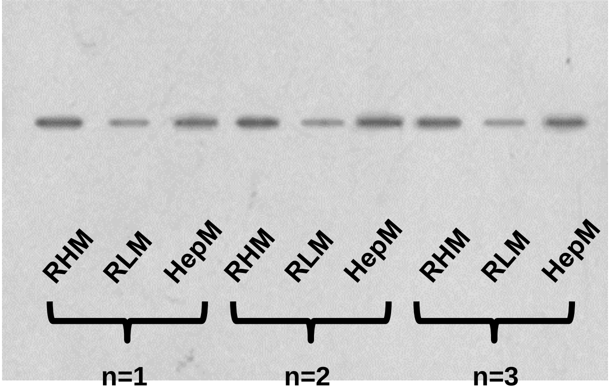

SDH 70 KDa

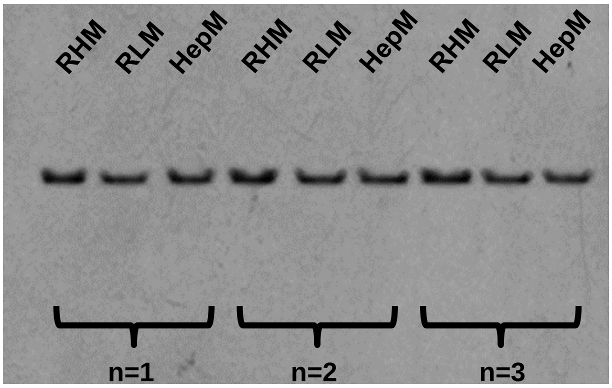

FH 46 KDa

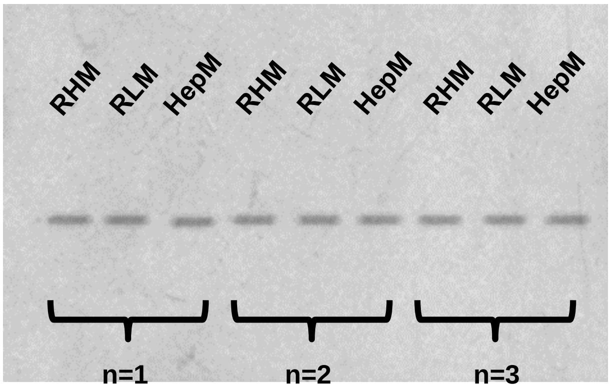

MDH 36 KDa

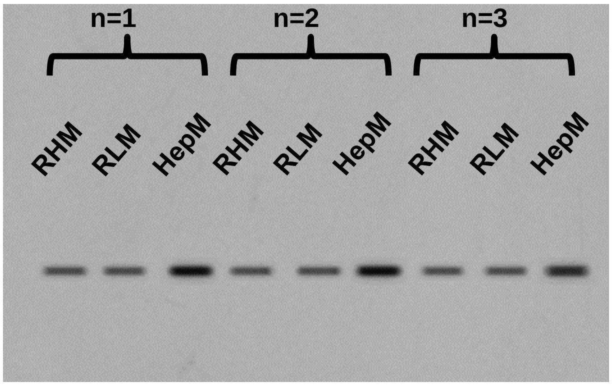

ND1 36 KDa

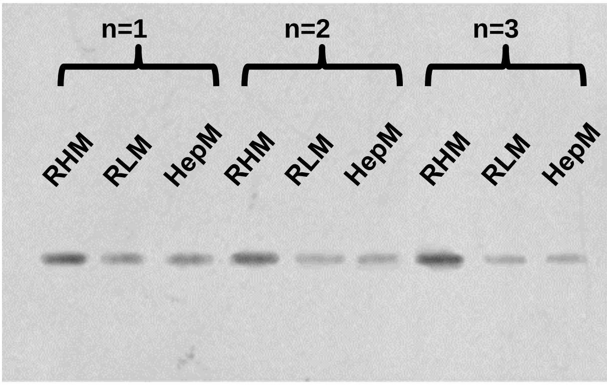

COX IV 17 KDa

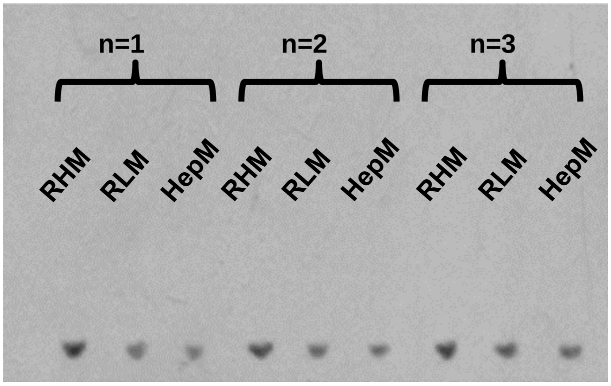

GDH 1/2 50 KDa

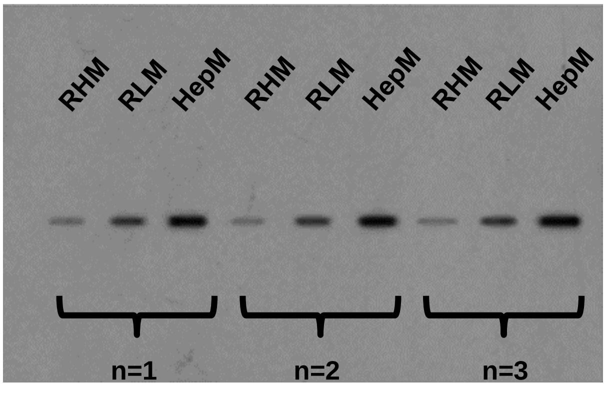

GLUTA 73 KDa

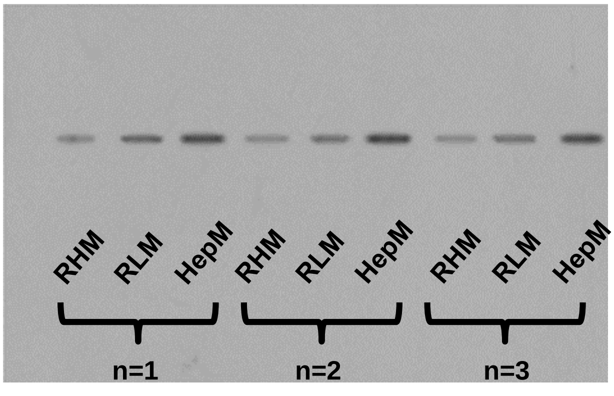

ME 64 KDa

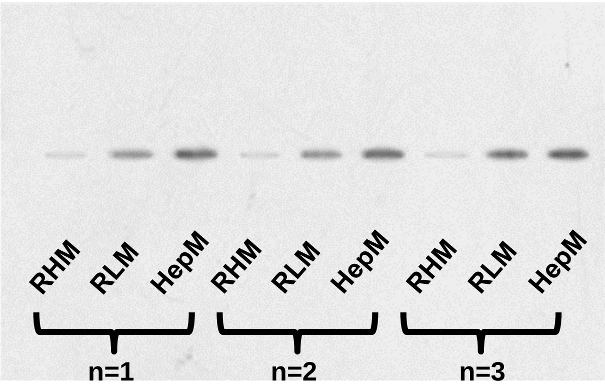

β-actin 43 kDa

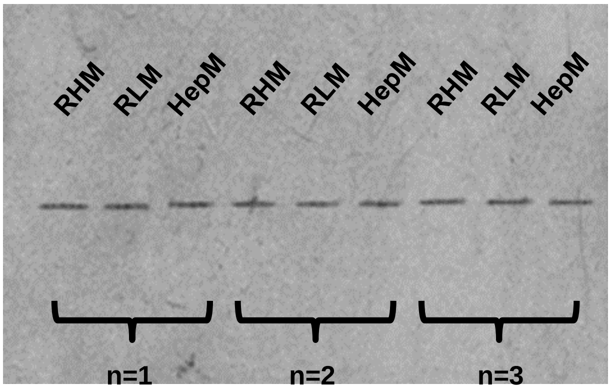

Supplement: Supplementary file 1 [file DataSheet1.pdf]
