# Supplementary material for: Modeling Krebs cycle from liver, heart and hepatoma mitochondria, supported Complex I as target for specific inhibition of cancer cell proliferation
Source: Front Oncol. 2025 Mar 26;15:1557638. doi: 10.3389/fonc.2025.1557638 (PMC11979947; doi:10.3389/fonc.2025.1557638)
Supplement: Supplementary file 2 [file DataSheet2.pdf]

Supplementary Material

Table S1. Summary of the kinetic parameter values used in the kinetic models of glucose central metabolism.

Enzyme	Parameters	HepM	RLM	RHM
PDH (Pyruvate dehydrogenase)	$Vmaxf$	0.06 [3]	0.0258 [3]	0.1 [3]
	Km_{Pyr}	0.21 [1]	0.21 [1]	0.2 [1]
	Km_{CoA}	0.014 [1]	0.014 [1]	0.04 [1]
	Km_{NAD+}	0.34 [1]	0.34 [1]	0.65 [1]
	$Keq\ (Keq/[CO_2])$	1.9×10^{9} [2]	1.9×10^{9} [2]	1.9×10^{9} [2]
	Km_{AcCoA}	10 [3]	10 [3]	10 [3]
	Km_{NADH}	10 [3]	10 [3]	10 [3]
CS (Citrate synthase)	$Vmaxf$	1.26 [1]	0.495 [1]	1.13 [1]
	Km_{AcCoA}	0.003 [1]	0.005 [1]	0.004 [1]
	Km_{OAA}	0.011 [1]	0.006 [1]	0.007 [1]
	Keq	2.24×10^{6} [4]	2.24×10^{6} [4]	2.24×10^{6} [4]
	Km_{CoA}	0.067 [5]	0.067 [5]	0.067 [5]
	$Km_{Citrate}$	1.6 [5]	1.6 [5]	1.6 [5]
ACO (Aconitase)	$Vmaxf$	0.359 [3]	0.095 [3]	0.173 [1]
	$Km_{Citrate}$	0.26 [1]	0.1 [1]	0.22 [1]
	Keq	10.8 [6]	10.8 [6]	10.8 [6]
	Km_{Iso}	0.12 [7]	0.12 [7]	0.12 [7]
IDH-NAD (Isocitrate dehydrogenase NAD+ dependent)	$Vmaxf$	0.125[1]	0.049 [1]	0.225 [1]
	Km_{Iso}	0.5 [1]	0.15 [1]	1.8 [1]
	Km_{NAD+}	0.25 [1]	0.45 [1]	0.2 [1]
	Keq (Keq/[CO2])	1479 [8]	1479 [8]	1479 [8]
	L	0.64 [1]	1.5 [1]	0.64 [1]
	$Ki_{NADH\ vs\ Iso}$	0.04 [1]	0.03 [1]	0.04 [1]
	Km_{NADH}	0.08 [1]	0.05 [1]	0.08 [3]
	$Km_{2\text{-}oxo}$	10 [3]	10 [3]	10 [3]
2-OGDH (α-ketoglutarate dehydrogenase)	$Vmaxf$	0.055 [3]	0.048 [3]	0.058 [1]
	$Km_{2\text{-}oxo}$	1 [1]	1.1 [1]	0.4 [1]
	Km_{AcCoA}	0.015 [1]	0.05 [1]	0.02 [1]
	Km_{NAD+}	0.4 [1]	0.4 [1]	0.5 [1]
	$Keq\ (Keq/[CO_2])$	1.6×10^{8} [2]	1.6×10^{8} [2]	1.6×10^{8} [2]
	Km_{SCoA}	10 [3]	10 [3]	10 [3]
	Km_{NADH}	10 [3]	10 [3]	10 [3]
SCS-A (Succinyl-CoA synthetase)	$Vmaxf$	0.2 [3]	0.087 [3]	0.229 [3]
	Km_{SCoA}	0.086 [9]	0.086 [9]	0.086 [9]
	Km_{ADP}	0.007 [9]	0.007 [9]	0.007 [9]
	Km_{Pi}	2.26 [9]	2.26 [9]	2.26 [9]
	$Vmaxr$	0.04 [3]	0.0179 [3]	0.0458 [3]
	Km_{ATP}	0.036 [9]	0.036 [9]	0.036 [9]

	Km_{CoASH}	0.036 [9]	0.036 [9]	0.036 [9]
	Km_{Suc}	0.49 [9]	0.49 [9]	0.49 [9]
SDH (Succinate dehydrogenase)	$Vmaxf$	0.356[3]	0.244 [3]	0.723 [3]
	Km_{Suc}	0.07 [1]	0.07 [1]	1 [1]
	Km_{CoQ}	0.48 [10]	0.48 [10]	0.48 [10]
	Keq	1.69 [10]	1.69 [10]	1.69 [10]
	Km_{QH2}	0.002 [10]	0.002 [10]	0.002 [10]
	Km_{Fum}	1.2 [10]	1.2 [10]	1.2 [10]
FH (Fumarase)	$Vmaxf$	3.356 [1]	0.172 [1]	0.527 [1]
	Km_{Fum}	1 [1]	0.53 [1]	0.26 [1]
	$Vmaxr$	0.34 [1]	0.187 [1]	0.523 [1]
	Km_{Mal}	0.4 [1]	1.7 [1]	0.3 [1]
MDH (Malate dehydrogenase)	$Vmaxf$	0.269 [1]	0.523 [1]	1.32 [1]
	Km_{Mal}	0.45 [1]	0.45 [1]	1.4 [1]
	Km_{NAD+}	0.11 [1]	0.11 [1]	0.2[1]
	$Vmaxr$	2.1 [1]	1.103 [1]	0.666 [1]
	Km_{NADH}	0.017 [1]	0.04 [1]	0.02 [1]
	Km_{OAA}	0.007 [1]	0.05 [1]	0.01 [1]
IDH-NADP (Isocitrate dehydrogenase NADP+ dependent)	$Vmax_f$	0.587[1]	0.108 [1]	1.059 [1]
	Km_{NADP}	0.078 [1]	0.0174 [11]	0.02 [1]
	Km_{Iso}	0.046 [1]	0.04 [1]	0.03 [1]
	Keq (Keq/[CO2])	1087 [11]	1087 [11]	1087 [11]
	Ki_{GSH}	15.5 [11]	23.6 [11]	22.3 [11]
	$Ki_{2\text{-}oxo}$	0.65 [11]	0.783 [11]	0.897 [11]
	Ki_{NADPH}	0.014 [11]	0.0147 [11]	0.0235 [11]
	Ki_{NAD}	2.98 [11]	2.98 [11]	2.98 [11]
ME (Malic enzyme)	$Vmaxf$	0.0065 [1]		0.07 [1]
	Km_{Mal}	1.1 [1]		1.4 [1]
	Km_{NADP^+}	0.4 [1]		0.5 [1]
	Keq (Keq/[CO2])	15.6 [12]		15.6 [12]
	Km_{Pyr}	10 [3]		10 [3]
	Km_{NADPH}	10 [3]		10 [3]
GDH (Glutamate dehydrogenase $NADP^+$ dependent)	$Vmaxf$	0.012 [11]	0.0735 [11]	0.002 [1]
	Km_{NADP}	0.491 [11]	0.491 [11]	0.53 [1]
	Km_{Glu}	5.2 [11]	5.2 [11]	8.8 [1]
	$Vmaxr$	0.159 [11]	0.544 [11]	0.019 [1]
	Km_{NADPH}	0.109 [13]	0.0566 [13]	0.109 [13]
	$Km_{2\text{-}oxo}$	0.733 [13]	0.371 [13]	0.08 [1]
	Km_{NH4}	10.3 [13]	18 [13]	10.3 [13]
	n	3.3 [13]	3.8 [13]	3.3 [13]
	L	31 [13]	5120 [13]	31 [13]
	$Ki\ GTP$	0.064 [13]	0.06 [13]	0.064 [13]
	$Ka\ ADP$	0.44 [13]	0.51 [13]	0.44 [13]
AST (Aspartate aminotransferase)	$Vmaxf$	0.527 [1]	0.42 [1]	4.3 [14]
	$Km_{2\text{-}oxo}$	1.3 [1]	1.3 [1]	2.5 [14]
	Km_{Asp}	0.6 [1]	0.6 [1]	1.3 [14]
	Keq	0.165 [15]	0.165 [15]	0.165 [15]

	Km $_{Glu}$<br>*Km* $_{OAA}$	10 [16]<br>0.25 [16]	10 [16]<br>0.25 [16]	10 [16]<br>0.25 [16]
GR (Glutathione reductase)	*Vmaxf*<br>*Km* $_{NADPH}$<br>*Km* $_{GSSG}$<br>*Keq*<br>*Km* $_{NADP}$<br>*Km* $_{GSH}$	0.024 [11]<br>0.009 [11]<br>0.053 [11]<br>64.1 [11]<br>0.9 [11]<br>3.8 [11]	0.037 [11]<br>0.009 [11]<br>0.046 [11]<br>64.1 [11]<br>0.9 [11]<br>3.8 [11]	0.010 [11]<br>0.009 [11]<br>0.046 [11]<br>64.1 [11]<br>0.9 [11]<br>3.8 [11]
NADH consumption	*Constant flux*	0.1965 [3]	0.1176 [3]	0.293 [3]
Pyr transporter	*Vmaxf*<br>*Km* $_{Pyr\ out}$<br>*Km* $_{Pyr}$<br>*Keq*	0.075 [3]<br>0.15 [19]<br>0.15 [19]<br>1 [3]	0.1 [3]<br>0.15 [19]<br>0.15 [19]<br>1 [3]	0.11 [3]<br>0.15 [19]<br>0.15 [19]<br>1 [3]
Mal-Suc transporter	*Vmaxf*<br>*Km* $_{Mal\ out}$<br>*Km* $_{Suc}$<br>*Keq*<br>*Km* $_{Mal}$<br>*Km* $_{Suc\ out}$	0.02 [3]<br>0.49 [22]<br>1.17 [22]<br>1 [3]<br>0.92 [22]<br>1.17 [22]	0.1 [3]<br>0.23 [22]<br>1.17 [22]<br>1 [3]<br>0.23 [22]<br>1.17 [22]	0.1 [3]<br>0.49 [22]<br>1.17 [22]<br>1 [3]<br>0.92 [22]<br>1.17 [22]
Mal-2oxo transporter	*Vmaxf*<br>*Km* $_{Mal\ out}$<br>*Km* $_{2\text{-}oxo}$<br>*Keq*<br>*Km* $_{Mal}$<br>*Km* $_{2\text{-}oxo\ out}$	0.007 [3]<br>1.4 [20]<br>0.046 [20]<br>1 [3]<br>1.4 [20]<br>0.046 [20]		0.76 [3]<br>1.4 [20]<br>0.046 [20]<br>1 [3]<br>1.4 [20]<br>0.046 [20]
Mal-Iso transporter	*Vmaxf*<br>*Km* $_{Mal\ out}$<br>*Km* $_{Iso}$<br>*Keq*<br>*Km* $_{Mal}$<br>*Km* $_{Iso\ out}$	0.14 [3]<br>0.8 [21]<br>0.08 [21]<br>1 [3]<br>0.8 [21]<br>0.08 [21]	0.04 [3]<br>0.8 [21]<br>0.08 [21]<br>1 [3]<br>0.8 [21]<br>0.08 [21]	0.65 [3]<br>0.8 [21]<br>0.08 [21]<br>1 [3]<br>0.8 [21]<br>0.08 [21]
ALT (Alanine transaminase)	*Vmaxf*<br>*Km* $_{alanine}$<br>*Km* $_{2\text{-}oxo}$<br>*Keq*<br>*Km* $_{Pyr}$<br>*Km* $_{Glu}$	0.327 [1]<br>10 [1]<br>2 [1]<br>0.7 [25]<br>0.9 [24]<br>15 [24]	0.056 [1]<br>9 [1]<br>1 [1]<br>0.7 [25]<br>0.9 [24]<br>15 [24]	
ROS	*V* $_{(constant\ flux\ irreversible)}$	0.019 [3]	0.015 [3]	0.009 [3]

Km in mM and *Vmax* in U/mg. For the RLM model, ME and Mal-2oxo transporter were not included. Meanwhile, the RHM model did not include ALT.

1 Values in range reported in Table 1.

[2] The *Keq* was calculated from the $\Delta G^{o\prime}$ = - 39.26 KJ mol^{-1} (PDH) (reported for physiological conditions, 37°C and pH=7, [Li et al., 2011]) and - 33 KJ mol-1 (2-OGDH) by using the equation $\Delta G^{o\prime}$= -RT *ln* Keq.
[3] arbitrary or adjusted value. These values were screened and selected when an optimum correspondence between model prediction and the experimental values (metabolites concentration and fluxes) were reached. PDH, ACO and 2OGDH activity values reported by Dietzen and Davis, 1993. SCS activity value adjusted in the range reported by Lambeth et al., 2004. SDH activity values adjusted to mitochondrial oxygen consumption in the presence of succinate plus rotenone.
[4] Value reported by Guynn et al., 1976.
[5] Values reported by Smith and Williamson, 1971.
[6] Value reported by Blair, 1969.
[7] Value reported by Guarriero-Bobyleva et al., 1973.
[8] Value reported by Moreno-Sánchez et al., 2014.
[9] Value reported by Johnson et al., 1998.
[10] Value reported by Wu et al., 2007.
[11] Value reported by Moreno-Sánchez et al., 2018.
[12] The *Keq* for malic enzyme reaction was taken from Veechm 1969. CO_2 concentration (2.2 mM) was incorporated into the *Keq* value and *Keq*/[CO_2] was calculated.
[13] Value reported by Moreno-Sánchez et al., 2020
[14] Value reported by Moreno-Sánchez et al., 2017.
[15] Value reported by Henson and Cleland, 1964.
[16] Value reported by Huynh et al., 1980.
[17] Value reported by Enander and Rydström, 1982.
[18] Value reported by Rydström et al., 1970.
[19] Value reported by Halestrap, 1975.
[20] Value reported by Palmieri et al., 1972.
[21] Value reported by Bisaccia et al., 1990.
[22] Value reported by Palmieri et al., 1971.
[23] Value reported by Swick et al., 1965.
[24] Value reported by Hopper and Segal, 1962.
[25] Value reported by Brosnan et al., 1970.

References

- Bisaccia F, De Palma A, Prezioso G, Palmieri F. Kinetic characterization of the reconstituted tricarboxylate carrier from rat liver mitochondria. Biochim Biophys Acta. 1990;1019(3):250-256. doi:10.1016/0005-2728(90)90201-e
- Blair JM. Magnesium and the aconitase equilibrium: determination of apparent stability constants of mangnesium substrate complexes from equilibrium data. Eur J Biochem. 1969, 8:287-91.
- Brosnan JT, Krebs HA, Williamson DH. Effects of ischaemia on metabolite concentrations in rat liver. Biochem J. 1970;117(1):91-96. doi:10.1042/bj1170091
- Dietzen DJ, Davis EJ. Oxidation of pyruvate, malate, citrate, and cytosolic reducing equivalents by AS-30D hepatoma mitochondria. Arch Biochem Biophys. 1993;305(1):91-102. doi:10.1006/abbi.1993.1397.

- Enander K, Rydström J. Energy-linked nicotinamide nucleotide transhydrogenase. Kinetics and regulation of purified and reconstituted transhydrogenase from beef heart mitochondria. J Biol Chem. 1982;257(24):14760-14766.
- Guarriero-Bobyleva V, Volpi-Becchi MA, Masini A. Parallel partial purification of cytoplasmic and mitochondrial aconitate hydratases from rat liver. Eur J Biochem. 1973, 34:455-8.
- Guynn RW, Gelberg HJ, Veech RL. Equilibrium constants of the malate dehydrogenase, citrate synthase, citrate lyase, and acetyl coenzyme A hydrolysis reactions under physiological conditions. J Biol Chem. 1973, 248:6957-65.
- Halestrap AP. The mitochondrial pyruvate carrier. Kinetics and specificity for substrates and inhibitors. Biochem J. 1975 Apr;148(1):85-96. doi: 10.1042/bj1480085. PMID: 1156402; PMCID: PMC1165509.
- Henson CP, Cleland WW. Kinetic studies of glutamic oxaloacetic transaminase isozymes. biochemistry. 1964, 3:338-45.
- Hopper S, Segal HL. Kinetic studies of rat liver glutamicalanine transaminase. J Biol Chem. 1962;237:3189-3195.
- Huynh QK, Sakakibara R, Watanabe T, Wada H. Glutamic oxaloacetic transaminase isozymes from rat liver. Purification and physicochemical characterization. J Biochem. 1980, 88:231-9.
- Johnson JD, Muhonen WW, Lambeth DO. Characterization of the ATP- and GTP-specific succinyl-CoA synthetases in pigeon. The enzymes incorporate the same alpha-subunit. J Biol Chem. 1998, 273:27573-9.
- Lambeth DO, Tews KN, Adkins S, Frohlich D, Milavetz BI. Expression of two succinyl-CoA synthetases with different nucleotide specificities in mammalian tissues. J. Biol. Chem. 2004; 279 36621–36624. https://doi.org/10.1074/jbc.M406884200.
- Li X, Wu F, Qi F, Beard DA. A database of thermodynamic properties of the reactions of glycolysis, the tricarboxylic acid cycle, and the pentose phosphate pathway. Database (Oxford). 2011 Apr 11;2011:bar005. doi: 10.1093/database/bar005.
- Moreno-Sánchez R, Marín-Hernández Á, Del Mazo-Monsalvo I, Saavedra E, Rodríguez-Enríquez S. Assessment of the low inhibitory specificity of oxamate, aminooxyacetate and dichloroacetate on cancer energy metabolism. Biochim Biophys Acta Gen Subj. 2017, 1861:3221-3236.
- Moreno-Sánchez R, Marín-Hernández Á, Gallardo-Pérez JC, et al. Physiological Role of Glutamate Dehydrogenase in Cancer Cells. Front Oncol. 2020;10:429. Published 2020 Apr 9. doi:10.3389/fonc.2020.00429
- Moreno-Sánchez R, Marín-Hernández Á, Gallardo-Pérez JC, Vázquez C, Rodríguez-Enríquez S, Saavedra E. Control of the NADPH supply and GSH recycling for oxidative stress management in hepatoma and liver mitochondria. Biochim Biophys Acta Bioenerg. 2018, 1859:1138-1150.
- Moreno-Sánchez R, Marín-Hernández A, Saavedra E, Pardo JP, Ralph SJ, Rodríguez-Enríquez S. Who controls the ATP supply in cancer cells? Biochemistry lessons to understand cancer energy metabolism. Int J Biochem Cell Biol. 2014, 50:10-23.
- Palmieri F, Prezioso G, Quagliariello E, Klingenberg M. Kinetic study of the dicarboxylate carrier in rat liver mitochondria. Eur J Biochem. 1971;22(1):66-74. doi:10.1111/j.1432-1033.1971.tb01515.x
- Palmieri F, Quagliariello E, Klingenberger M. Kinetics and specificity of the oxoglutarate carrier in rat-liver mitochondria. Eur J Biochem. 1972;29(3):408-416. doi:10.1111/j.1432-1033.1972.tb02003.x

- Rydström J, da Cruz AT, Ernster L. Factors governing the kinetics and steady state of the mitochondrial nicotinamide nucleotide transhydrogenase system. Eur J Biochem. 1970;17(1):56-62. doi:10.1111/j.1432-1033.1970.tb01133.x
- Smith CM, Williamson JR. Inhibition of citrate synthase by succinyl-CoA and other metabolites. FEBS Lett. 1971 Oct 15;18(1):35-38. doi: 10.1016/0014-5793(71)80400-3.
- Swick RW, Barnstein PL, Stange JL. The metabolism of mitochondrial proteins. i. distribution and characterization of the isozymes of alanine aminotransferase in rat liver. J Biol Chem. 1965;240:3334-3340.
- Wu F, Yang F, Vinnakota KC, Beard DA. Computer modeling of mitochondrial tricarboxylic acid cycle, oxidative phosphorylation, metabolite transport, and electrophysiology. J Biol Chem. 2007 Aug 24;282(34):24525-37. doi: 10.1074/jbc.M701024200. Epub 2007 Jun 25. PMID: 17591785.

Table S2. Fixed metabolites concentrations used in the kinetic models

Metabolite (mM)	HepM	RLM	RHM
ADP	0.8 ± 0.6 (3)[1]	1.7[2]	1.3 ± 0.22 (3)[1]
Pi	5 [2]	5 [2]	5 [2]
ATP	2.5 (2)[1]	1.8 (2)[1]	8.5 (2)[1]
Aspartate	0.9 (2)[1]	2 ± 3 (3)[1]	2.3 ± 1.1 (3)[1]
Glutamate	0.3 (2)[1]	0.15 ± 0.1 (3)[1]	0.5 ± 0.7 (3)[1]
NH_4	0.001[2]	0.2[2]	0.001[2]
GTP	0.14 [3]	0.17 [3]	0.17 [3]
Pyr_{out}	2	2	2
Mal_{out}	5	5	5
Suc_{out}	0.3 ± 0.3 (3)[1]	0.27 (2)[1]	0.001[2]
$2OG_{out}$	0.14 (2)[1]	*	0.15 ± 0.01 (3)[1]
CoQ	0.97 [4]	0.97 [4]	0.97 [4]
QH_2	0.38 [4]	0.38 [4]	0.38 [4]
$Isocitrate_{out}$	0.32 ± 0.27 (3)[1]	0.55 ± 0.21 (5)[1]	0.001[2]
Alanine	0.2 (2)[1]	4 ± 0.21 (3)[1]	*

*This metabolite was not considered in the model.

1. Metabolite concentration determined in this study. Amino acids were determined according to the method described in supplementary material.
2. Value adjusted in the interval reported.
3. Moreno-Sánchez R, Marín-Hernández Á, Gallardo-Pérez JC, et al. Physiological Role of Glutamate Dehydrogenase in Cancer Cells. *Front Oncol*. 2020;10:429. Published 2020 Apr 9. doi:10.3389/fonc.2020.00429.
4. Wu F, Yang F, Vinnakota KC, Beard DA. Computer modeling of mitochondrial tricarboxylic acid cycle, oxidative phosphorylation, metabolite transport, and electrophysiology. J Biol Chem. 2007 Aug 24;282(34):24525-37. doi: 10.1074/jbc.M701024200. Epub 2007 Jun 25. PMID: 17591785.

Determination of amino acids

The mitochondrial neutralized acid extracts were centrifuged at 16200 x g for 5 min to remove the $KClO_4$ salts. For derivatization, 20 µl of the supernatant was recovered and derivatized with 50% o-phthalaldehyde solution (37 mM o-phthalaldehyde, 5% methanol, 5% β -mercaptoethanol dissolved in 0.4 M boric acid, pH 9.9), and stirred for 1 min. The reaction was stopped by dilution with 0.5 mL of 20 mM NaH_2PO_4 pH 7.8 and filtered through a Millex-Millipore filter (0.45 µm pore diameter).

A filtered aliquot was injected in a UPLC apparatus coupled to a fluorescence detector (Waters 1525/2475, Milford, MA, USA) for amino acids separation, using a reverse phase column with 1.7 µm particle size and 2.1 x 50 mm (Waters 186002350, Milford, MA, USA). The column was equilibrated with a 20 mM NaH2PO4 pH 7.8 solution, using a flow rate of 0.4 mL/min. Amino acids were separated by applying a linear gradient of a solution of acetonitrile: methanol: water (30:20:20) and detected by fluorescence (340 nm excitation, 460 nm emission). The amino acids were identified by the retention time and their quantification was performed by the area under the curve of their respective standards as references, which were previously determined under the same conditions as the samples [1,2].

[1] J.D. García-García, K.A. Peña-Sanabria, R. Sánchez-Thomas, R. Moreno-Sánchez, Nickel accumulation by the green algae-like Euglena gracilis, J. Hazard. Mater. 343 (2018) 10–18. https://doi.org/10.1016/j.jhazmat.2017.09.008.
[2] B.J. Meussen, A.N.T. van Zeeland, M.E. Bruins, J.P.M. Sanders, A Fast and Accurate UPLC Method for Analysis of Proteinogenic Amino Acids, Food Anal. Methods. 7 (2014) 1047–1055. https://doi.org/10.1007/s12161-013-9712-7.

Table S3. Steady-state fluxes obtained by kinetic modeling for the enzymes of the KC pathway.

Enzyme/transporter/system	HepM	RLM	RHM
CS	56.2	24	88.4
ACO	56.2	24	88.4
IDH^{NAD+}	24.3	6.4	1.5
2OGDH	50.5	41.8	49.6
SCS	50.5	41.8	49.6
SDH	46	14.4	15.3
FUM	46	14.4	15.3
MDH	65.5	45.2	153.4
PDH	56.2	24	88.4
AST	-9.2	-21.1	-65
ALT	-1.2	0.77	*
GDH	0.27	0.77	8×10^{-6}
ME	1.5	*	8×10^{-6}
NADH consumption	196.5	117.6	293
GR	19	15	9
ROS	19	15	9
IDH^{NADP}	17.2	14.2	8.9
Pyr transport	55.9	23.2	88.4
Mal/Suc trasnporter	4.5	27.4	34.2
Mal/Iso transporter	14.6	3.3	77.9
Mal/2oxo transport	1.78	*	25.9

Fluxes in nmol/min*mg of protein. *These reactions do not have values because they were not considered in the corresponding kinetic models.
